# Eco-Friendly Carboxymethyl Cellulose-Functionalized Binary (ZnS, In_2_S_3_) and Ternary (Zn–In–S) Quantum Dot Nanophotocatalysts for the Advanced Photodegradation of Organic Dye Pollutants in Water

**DOI:** 10.1021/acsomega.5c12542

**Published:** 2026-07-01

**Authors:** Thiago Luís de Souza Esteves, Alexandra A. P. Mansur, Isadora C. Carvalho, Herman S. Mansur

**Affiliations:** Center of Nanoscience, Nanotechnology, and Innovation—CeNano2I, Department of Metallurgical and Materials Engineering, 28114 Federal University of Minas Gerais, UFMG, Av. Pres. Antônio Carlos, 6627— Engineering School, 31.270-901 Belo Horizonte, Minas Gerais, Brazil

## Abstract

Rapid urbanization and industrialization have led to severe water contamination, particularly from irresponsible discharges or accidental spills of synthetic dyes. Environmental nanotechnology, specifically semiconductor quantum dots (QDs), offers disruptive solutions for the photocatalytic degradation of organic pollutants. In this study, binary (ZnS and In_2_S_3_) and ternary (Zn–In–S) QDs functionalized with carboxymethyl cellulose (CMC) were synthesized via a sustainable, aqueous colloidal route. The CMC biopolymer served as a macromolecular template and stabilizing ligand for the nucleation and controlled growth of the QD. As nanocatalysts, they were evaluated for the photodegradation of methylene blue (MB, cationic) and methyl orange (MO, anionic) model dyes under UV irradiation across various pH conditions (3.0, 5.0, and 7.0). The results confirmed the formation of colloidal nanocrystalline QDs stabilized by CMC, with an average size of approximately 3 nm and optical properties dependent on the core chemical composition. Photocatalytic assays demonstrated removal efficiencies of 30–70%, with optimal degradation at pH 5.0, following the pseudo-second-order model. Hence, these findings demonstrate that CMC-stabilized QDs can provide a sustainable, potentially scalable nanoplatform for advanced oxidative treatment of polluted water.

## Graphical abstract

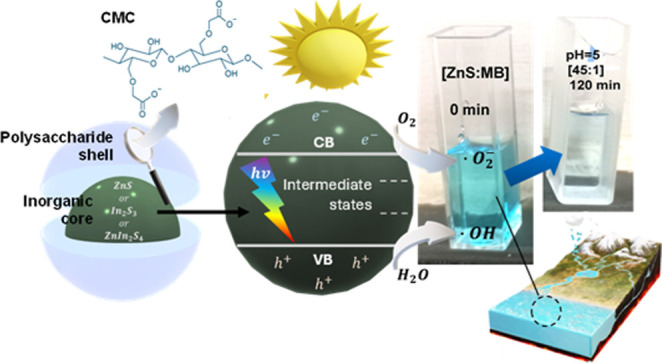

## 1 Introduction

Since the late 20th century, nanoscience and nanotechnology have emerged as disruptive fields, and the world has begun to recognize their potential to advance human life.^1^ It is estimated that the global nanomaterials market, which was approximately $7.1 billion in 2020, will grow at an average annual rate of 9.7% to reach $12.1 billion by 2026. As a result, various types of nanomaterials and nanostructures can be utilized across multiple industries to address common challenges.^1,2^ Nanomaterials are currently widely used in technology and engineering due to their unique properties, which enable high-quality displays, faster computers, enhanced material properties, innovative solutions for renewable and sustainable energy, as well as novel, sophisticated applications in biomedical fields, biosensors, and nanomedicine.^3–7^

In this regard, the environmental industry has leveraged nanomaterials by investing millions of dollars in energy storage, air, soil, and water decontamination, among other applications.^8^ In addition to irresponsible discharges of industrial and domestic effluents into water bodies, environmental disasters involving toxic dyes have underscored the urgent need to investigate these problems and to propose new solutions to prevent and remediate accidental spills.^9–15^

Dyes are commonly aromatic organic molecules applied to substrates to impart permanent color that resists fading upon exposure to water, light, oxidizing agents, and microbial attack.^8,12–15^ Due to these advantages, various dyes are used across industries such as textiles, food, rubber, printing, cosmetics, medicine, plastics, concrete, and the paper industry for multiple purposes, accounting for approximately 17–20% of water pollution.^10–12^ Textile manufacturing is the most dye-consuming sector, using highly complex compounds with diverse structural groups. Some of the most common are methylene blue (MB), a cationic dye, and methyl orange (MO), an anionic dye.^13,16^ These dyes, above certain threshold levels, are recalcitrant (i.e., low degradability), persisting, and accumulating in the environment, which can disrupt aquatic ecosystems. Such contaminated water bodies become toxic to living organisms and yield nonpotable water unsafe for human consumption. Also, it is worth noting that the MB and MO are harmful to human health at high concentrations, as they are carcinogenic and mutagenic, posing a serious threat through exposure to polluted water sources or food chains.^8,13–15^

Dye degradation mediated by nanomaterials occurs primarily through bond-breaking and chemical modification, yielding less complex byproducts with lower toxicity, or, ideally, nontoxic derivatives. In this scenario, advanced oxidation processes (AOPs) have garnered significant attention for their numerous advantages, particularly their environmental friendliness. They typically offer high efficiency associated with the capability to degrade organic contaminants (e.g., dyes, pharmaceuticals, drugs).^12,17–20^ Among the various AOPs, including Fenton’s reagent, ozonation, electrochemical oxidation, and advanced oxidation using peroxides, photocatalytic technology stands out for its unique set of advantages and cost-effectiveness.^12,17,21^ In line with this strategy to provide innovative tools for environmental remediation, novel nanomaterials have been developed.^21,22^ Quantum dots (QDs) are a class of nanomaterials that, owing to their remarkable semiconductor properties, can be used to remove dyes from water via multiple advanced processes.^21,22^ Basically, QDs are zero-dimensional semiconductor nanocrystals with size-dependent properties resulting from quantum confinement. When the nanoparticle size is smaller than the Bohr radius (*a*_B_) parameter, it behaves like a potential well, limiting the space available for the coupled electron–hole pair (h^+^/e^–^, called an exciton), which results in a larger bandgap for the semiconductor nanoparticle compared to the bulk analog.^13–15,21–23^ This unique optoelectronic characteristic is fascinating and can promote several processes and chemical reactions. It offers high potential for various environmentally driven applications, such as decontaminating water bodies by promoting bond-breaking of organic pollutants into less toxic intermediates and molecules. Basically, the photoinduced degradation mechanism involves an electron (e^–^) being excited from the valence band (VB) to the conduction band (CB) by incident photons, thereby creating a hole (h^+^) in the valence band.^13–15,21,22^ Consequently, there is a strong tendency for recombination between the electron and the hole, which competes with other species in the medium, such as electron donors (e.g., water, oxygen, and hydroxyl radicals), during dynamic oxidation or reduction processes. However, to be effective, the charge-separation process should outpace recombination, promoting interfacial interactions and generating reactive oxygen species (ROS) that can oxidize or decompose nearby organic compounds, including dyes and pigments.^22^ Moreover, these nanomaterials possess a large surface area that favors adsorption and accelerates these processes at heterogeneous solid–liquid interfaces, as these mechanisms rely on the proximity of the nanoparticles to the dye.^21,24^

Given the need to synthesize QDs sustainably, predominantly in aqueous media, this remains a significant challenge and is still under intensive development. Initially, QDs were mostly produced using heavy transition-metal sulfide compounds (e.g., CdS, PbS, HgS), chemical surfactants, and organic ligands, which limited their applications due to the high toxicity of these materials.^25–30^ Recently, efforts have emerged to develop green synthesis and eco-friendly approaches for the use of QDs in environmental and biomedical applications.

In this context, biopolymers such as polysaccharides (e.g., chitosan, carboxymethyl cellulose, and hyaluronic acid) and peptides have been investigated as biomolecules for the creation of functionalized hybrid nanosystems.^28–30^ Polysaccharides, particularly carboxymethyl cellulose (CMC), have been used to stabilize nanomaterial size and produce nontoxic QDs, such as zinc sulfide (ZnS) and indium sulfide (In_2_S_3_),^21,22,30–32^ in numerous environmental and biomedical applications via aqueous colloidal routes.^21,22,33^ These polysaccharides are sustainable, biocompatible, and water-soluble polymers for synthesizing QDs for environmental applications, including photocatalysis.^21,22^

Both ZnS and In_2_S_3_ are binary systems, meaning they are two-component semiconductor nanocrystals (Metal = Zn^2+^ or In^3+^, and S^2–^ counterion). ZnS is a II–IV semiconductor with a 3.62 eV bulk bandgap and a 5.5 nm Bohr radius; thus, it can be considered a “wide bandgap” semiconductor, with the nanoparticle bandgap depending on its size.^21,22,31,34^ On the other hand, In_2_S_3_ is considered a “narrow bandgap” semiconductor with a bulk bandgap of 2.3 eV and a Bohr radius that reaches 30 nm.^13,15,35–38^ These materials can also be combined with other compounds at the nanoscale (e.g., metal sulfides, TiO_2_, InP, nanometals, etc.) to improve their properties and create innovative nanoarchitectures.^17,39^ Furthermore, these semiconductor nanoparticles allow modification of the bandgap and energy structure by selecting the composition and/or stoichiometry of their precursors, making the Zn–In–S (ZIS) ternary QD nanosystems an opportunity to engineer their optoelectronic properties as well as their potential catalytic performance. Thus, designing an appropriate bandgap structure of nanosized semiconductors could significantly enhance the generation of chemical radicals (h^+^/VB + H_2_OIII ^•^OH and e^–^/CB + O_2_ III ^•^O_2_^–^) while also increasing their absorption in the abundant and renewable sunlight, including the visible range (∼44% of the solar spectrum). Additionally, the properties of the heterojunction and the surface chemistry of these QDs can be tailored and combined with biopolymers (e.g., CMC as functional capping ligands) to interact specifically with pollutants, while providing biocompatibility and environmental friendliness.^21,22^ Hence, the application of QDs to develop innovative AOP alternatives based on heterogeneous photocatalysis can significantly address some current challenges in remediating dye pollutants in water bodies and mitigate their harmful environmental impact.^12,21,22^

In this view, this study reports the design, development, synthesis, and extensive characterization of binary QDs composed of ZnS and In_2_S_3_, as well as ternary Zn–In–S (ZIS) nanoalloys, chemically produced via an aqueous green-chemistry colloidal route under mild conditions. CMC polysaccharide was used as both a stabilizing ligand and a macromolecular template for nucleation and controlled growth of an inorganic semiconductor core, thereby yielding optically active, functionalized, hybrid inorganic–organic quantum dot-bearing nanosystems with colloidal stability, eco-friendliness, biocompatibility, and an affinity moiety for photocatalytic processes. These nanomaterials demonstrated photocatalytic activity for the degradation of anionic and cationic dye models mimicking organic pollutants in water.

## 2 Experimental Section

### 2.1 Materials

Zinc chloride (ZnCl_2_ > 98%, CAS: 7646-85-7), Sodium sulfide nonahydrate (Na_2_S·9H_2_O > 98%, CAS: 1313-82-2), Indium nitrate (In­(NO_3_)_3_ ·*x*H_2_O, In > 28.5%, CAS: 2007398-97-8), Sodium carboxymethyl cellulose (molecular weight, *M*_w_ = 250.000 g/L, degree of substitution, DS = 0.77, CAS: 9004-32-4), and Methylene blue hydrate (MB·*x*H_2_O, ≥95%, CAS: 122965-43-9) were supplied by Sigma-Aldrich, USA.

Hydrochloric acid (HCl, 37%) and Methyl orange (MO, 85%, CAS: 547-58-0) were procured by Synth, Brazil. Additionally, deionized water with a resistivity ≥18 MΩ·cm was used, as provided by Water Purification Systems (Millipore Simplicity, USA).

### 2.2 ZnS Quantum Dots Synthesis

To avoid redundancy with the synthesis process reported by our group in the literature,^21,22^ only the new procedures will be described in detail in the Experimental Section. The ZnS quantum dot synthesis protocol was adapted from the methodology of Caires et al.^31^ Initially, precursor solutions of carboxymethyl cellulose (CMC) at 0.2 wt %, three ZnCl_2_ solutions at concentrations of 9 × 10^–3^ mol/L, 9 × 10^–4^ mol/L, and 1 × 10^–4^ mol/L, and Na_2_S at 5 × 10^–2^ mol/L were prepared at room temperature using deionized water. Under moderate stirring, 25 mL of the CMC solution and 10 mL of each ZnCl_2_ solution were added to a flask. Stirring was maintained for 10 min, and the solution was stored at a temperature between 4 and 6 °C for 24 h. Then, under moderate stirring, 2 mL of the Na_2_S solution was added dropwise. Stirring was maintained for 30 min at the end of the process. The suspension of QDs formed was then stored in the dark at a temperature between 4 and 6 °C.

### 2.3 In_2_S_3_ Quantum Dots Synthesis

The In_2_S_3_ QD synthesis protocol was adapted from the methodology of Mansur et al.^40^ Initially, precursor solutions of CMC at 1 wt %, In­(NO_3_)_3_ at 1 × 10^–2^ mol/L, and Na_2_S at 1 × 10^–2^ mol/L were prepared at room temperature using deionized water. Under moderate stirring, 2 mL of the carboxymethyl cellulose solution and 45 mL of deionized water were added to a flask. Then, 2 mL of the In­(NO_3_)_3_ solution and 3 mL of Na_2_S were added, both dropwise. Stirring was maintained for 60 min, and the QD suspension was stored in the dark at a temperature between 4 and 6 °C at the end of the process.

### 2.4 Zn–In–S Quantum Dots Synthesis

The Zn–In–S QD synthesis protocol was adapted from the methodology of Zikalala et al.^41^ Initially, precursor solutions were prepared at room temperature using deionized water, with the following concentrations: CMC at 1 wt %, ZnCl_2_ at 3 × 10^–2^ mol/L, In­(NO_3_)_3_ at 3 × 10^–2^ mol/L, and Na_2_S at 6 × 10^–2^ mol/L.

Under moderate stirring, 2 mL of the CMC solution and 43 mL of deionized water were added. 0.5 mL of the ZnCl_2_ solution, 1 mL of the In­(NO_3_)_3_ solution and 1 mL of the Na_2_S solution were added. Finally, the solution was refluxed at 95 °C with moderate stirring for 60 min. After the solution cooled, it was stored in the dark at a temperature between 4 and 6 °C. QDs were synthesized in the proportion 1:2:4 (Zn/In/S).

### 2.5 Characterization of Nanostructures and Degradation Products

All measurements, unless specified, were conducted at room temperature (RT).

The absorption properties of quantum dots, as optically active nanomaterials, were measured by ultraviolet–visible spectroscopy (UV–vis) using a Lambda EZ-210 (PerkinElmer) spectrometer, in transmission mode, scanning from 190 to 600 nm with a quartz cuvette.

Emission spectra were measured by FluoroMax-Plus-CP (Horiba Scientific) based on steady-state spectroscopy (λ_excitation_ = 300 nm). Additionally, the 3D excitation–emission contour maps of these nanosystems were collected (λ_excitation_ = 200 to 500 nm; λ_emission_ = 300 to 600 nm; and slit = 2.5 nm).

Morphological characteristics and size distribution of inorganic nanoparticles capped by CMC ligands were evaluated from high-resolution transmission electron microscopy (HR-TEM, 200 kV, Tecnai G2-20-FEI, FEI Company) associated with an energy-dispersive X-ray spectroscopy analyzer (EDX, EDAX detector integrated to HR-TEM) for semiquantitative evaluation of elemental composition. Samples were concentrated by filtering quantum dot solutions through an Amicon Ultra Filter (Millipore, USA) with a 100 kDa cutoff cellulose membrane, then washed three times with deionized water before placing each suspension onto a holey carbon-supported copper grid. The QD size and size distribution data were assumed to be the average of 50 random measurements.

Zeta potential (ζ-potential or ZP, electrokinetic potential) and dynamic light scattering (DLS) analyses were performed using the ZetaPlus Instrument (Brookhaven Instruments Corporation—red diode laser 35 mW and λ = 660 nm, angle 90°) at a minimum of 3 runs of 3 replicates (*n* ≥ 9).

Chemical composition was evaluated by X-ray fluorescence (XRF) Supermini 200 (Rigaku, Japan, Software ZSX, 50 kV, current of 4 mA, and 200 W).

Surface characterization was performed via X-ray photoelectron spectroscopy (XPS) using Mg Kα radiation within a Kratos Analytical Amicus instrument. To analyze QD inorganic cores beneath the immediate surface, the samples underwent Ar^+^ ion etching for 3 s. Data processing included aligning the binding energy to the C 1s peak at 285 eV. For sample preparation, colloidal dispersions were cast onto aluminum substrates and dried at 40 ± 1 °C to remove the water medium, yielding a stable film. The XPS technique was also used to evaluate the formation of degradation products and intermediates on the sample surface (without Ar^+^ etching).

Samples were prepared by casting suspension concentrates (ZnS@CMC, ZnS@CMC + MB adsorbed, and ZnS@CMC + MB after 120 min of irradiation) obtained by centrifugation (Amicon Ultra Filter with a 50 kDa cutoff cellulose membrane) in plastic molds.

A Nicolet 6700 spectrometer (Thermo Fisher Scientific) was used to collect Fourier transform infrared (FTIR) spectra via the attenuated total reflectance (ATR) technique.

Data were recorded from 4000 to 800 cm^–1^ at a resolution of 4 cm^–1^ over 32 scans, incorporating automatic background subtraction.

### 2.6 Photocatalytic Experiments Based on Dye Pollutant Models—MB and MO

Two model organic pollutants were used for the photocatalysis assays: methylene blue (MB), a cationic dye, and methyl orange (MO), an anionic dye. Thus, five systems were evaluated: 2 systems using ZnS QDs stabilized by CMC nanophotocatalysts (ZnS@CMC) were tested for degrading MB and MO; 2 systems using In_2_S_3_ QD functionalized with CMC nanophotocatalysts (In_2_S_3_@CMC) for degrading MB and MO; and 1 system using Zn–In–S QDs functionalized with CMC (ZIS@CMC) in the proportion [1:2:4] nanophotocatalysts to the model MB dye for degrading. The ZnS/MB systems were analyzed at three pH conditions, acidic (pH = 3.1 ± 0.2), mildly acidic (pH = 5.1 ± 0.2), and neutral (pH = 7.1 ± 0.2), using two [QD/dye] mass ratios, [45:1] and [23:1]. Analogously, the ZnS/MO systems were tested under the same pH conditions detailed above, but with a single [QD/dye] mass ratio of [23:1]. Regarding the In_2_S_3_@CMC photocatalysts, the systems In_2_S_3_/MB and In_2_S_3_/MO were evaluated under mildly acidic conditions (pH = 5.1 ± 0.2), using the mass ratio [QD/dye] of [10:1].

For ZIS@CMC photocatalysts, the system evaluated MB dye at a [23:1] mass ratio at two pH values (7.0 ± 0.5 and 5.1 ± 0.2). The concentration of the model dye (MB or MO) was maintained at 2.5 mg/L in the initial solution before catalysis (i.e., at *t*_0_). In comparison, the concentration of QDs was varied to test different dye ratios for photocatalysis: 113 mg/L [45:1], 56.5 mg/L [23:1], and 25 mg/L [10:1]. For this purpose, the QD solutions synthesized in this work and an MB hydrate solution (MB·*x*H_2_O, 16.1% H_2_O) at a concentration of 8.9 × 10^–2^ g/L were used.

To ensure reproducibility and a standardized comparison of photocatalytic performance, the ratio between the nanophotocatalyst and the organic dye (MB or MO) was determined based on the theoretical mass of the respective inorganic semiconductor cores. The total mass of the active photocatalyst was derived from the stoichiometric amounts of the metal of the binary ZnS, In_2_S_3,_ and ternary Zn–In–S systems. For these calculations, a quantitative conversion (i.e., 100% yield) was assumed for the reaction of the metallic cations, Zn^2+^ and In^3+^, with the sulfide precursor (S^2–^), supported by their extremely low values for solubility product constants (K_sp,_ ZnS ∼ 1.6 × 10^–24^, and In_2_S_3_ ∼ 6.3 × 10^–36^).^42^ Based on this theoretical concentration, the volume of QD suspension to be added was adjusted to achieve the desired photocatalytic (QD/dye) solution dosage.

The solution was then placed in a 9 cm-diameter Petri dish for the photocatalysis test. The Petri dish was positioned inside a dark chamber, parallel to the excitation light source, an ultraviolet-C lamp (UV–C, 6 W, λ_excitation_ = 254 nm, Boitton Instruments) at a vertical distance of 20 cm.

The photocatalysis was evaluated at room temperature for 120 min, with 2 mL aliquots collected at 0, 2, 5, 10, 15, 30, 60, and 120 min.

As the reacting medium was collected, the MB concentration was determined by UV–vis spectroscopy, monitoring the absorbance maxima of MB and MO (λ_max_ = 664 and 464 nm, respectively). During this time, the light was turned off, and the stopwatch was paused.

The sample collected from the solution medium was immediately returned to the Petri dish after analysis, and the photocatalysis assay was restarted. This procedure was repeated until 120 min had elapsed. The duration of the photocatalysis experiment was selected based on preliminary “screening” studies that showed the nanosystems typically reached a “degradation plateau” after about 100–110 min had elapsed.

## 3 Results and Discussion

### 3.1 Synthesis and Characterization of Quantum Dots

The controlled synthesis of CMC-QD nanostructures significantly determines most of their optoelectronic properties and, therefore, their photocatalytic behavior. Thus, QDs were synthesized via an aqueous colloidal “bottom-up” coprecipitation route in the presence of CMC polysaccharide, offering scalability and high yield while forming a water-dispersible suspension. This CMC-templated procedure controls QD nucleation and growth (regulating nanoscale dimensions within “quantum confinement regime”), prevents nanoparticle agglomeration, and tailors their surface characteristics. The synthesis pathway can be divided into two major steps: (A) Complexation and (B) Coprecipitation. At the first step, after each precursor was solubilized in water, metal salt solutions were added to the previously prepared CMC solution. Considering that CMC is in its negative form (anionic) throughout all synthesis steps (pH 7.0 > p*K*_a_ ∼ 4.5), the cationic species (Zn^2+^ and/or In^3+^) form complexes with the CMC biopolymer (R–COO^–^=M^*x*+^). In the sequence, in the second stage, the sodium sulfide solution (S^2–^) was injected into the flask, resulting in the immediate formation of embryos of M_*y*_S_*x*_ QDs nuclei (“burst nucleation”), as a result of the supersaturation of the precursors, much superior to the extremely low *K*_sp_ (ZnS ∼ 10^–24^, and In_2_S_3_ ∼ 6.3 × 10^–36^).^42^ This causes a rapid depletion of the concentration of reagents in solution, decelerating the nucleation process. That is followed by the slow growth of the QD nanocrystals, during which the negatively charged CMC polymeric capping controls reaction kinetics and prevents agglomeration through chain repulsion and steric hindrance. Thus, these multiple steps result in the chemical stabilization and functionalization of optically active ultrasmall QDs@CMC nanohybrids. The following sections present the comprehensive characterization and performance results of each QD@CMC noncolloidal system (i.e., binary and ternary QDs).

#### 3.1.1 Characterization of ZnS QD/CMC Nanocolloids—ZnS@CMC

For light-harvesting photocatalytic applications, the combination of wide- and medium-bandgap semiconductor materials is particularly interesting for capturing a broader range of the solar spectrum. In this sense, ZnS, a wide-band gap semiconductor, is nontoxic and environmentally benign, and is suitable for capturing UV light.^21^

Thus, the ultraviolet absorption spectroscopy (UV–vis) and photoluminescence spectroscopy (PL) characterizations of the ZnS@CMC nanoparticles were performed to investigate their optical activity, specifically the absorption and emission properties, with the results displayed in Figure 1. These properties are of paramount relevance for assessing the key characteristics of semiconductors and their energy band structure.

**Figure 1:**
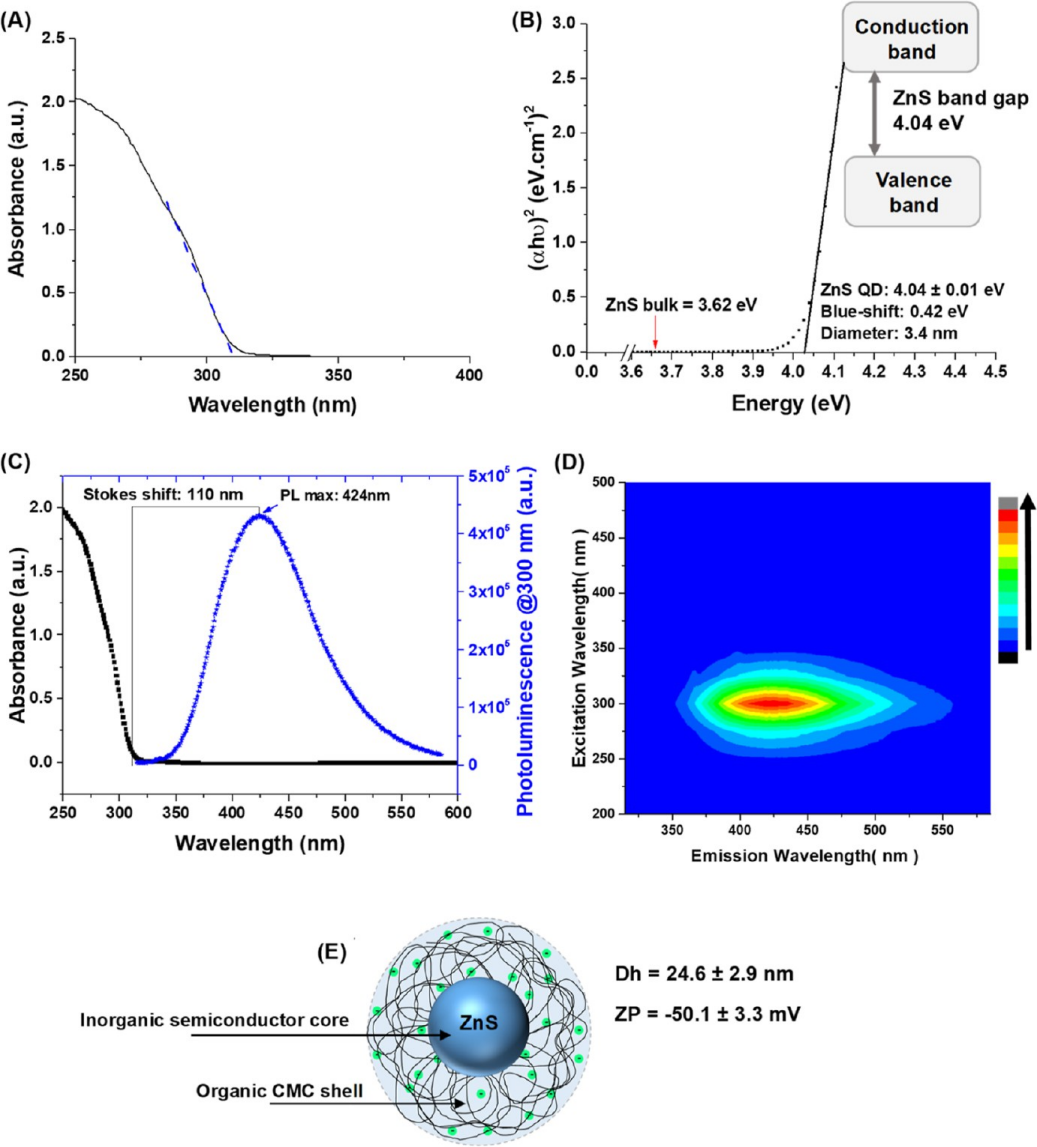
(A) UV–vis spectra of synthesized ZnS@CMC QDs. (B) Tauc plot with a schematic representation of the bandgap energy structure. (C) Absorbance and photoluminescence spectra. (D) 3D excitation/emission spectra. (E) Scheme of size and superficial charge of colloids showing the ZnS@CMC hybrid organic–inorganic structure of nanophotocatalyst (not to scale).

Quantum dots, as semiconductor nanomaterials, exhibit strong absorption at a specific wavelength, which should result in an energy “blueshift” relative to their bulk counterparts.^23^ For the ZnS QDs produced, there was a steep increase in absorption in the region of 250–300 nm, and the onset was around 310 nm (Figure 1A). In addition, the Tauc’s plot (Figure 1B), which relates absorption to electronic transitions in semiconductors, was used to determine the band gap of these nanomaterials.^43^ Thus, the band gap of ZnS QDs was estimated as 4.04 ± 0.01 eV, which represents an energy “blueshift” of approximately 0.4 eV from bulk ZnS (*E*_g_ = 3.6–3.7 eV) reported in the literature.^21^ These findings confirmed the formation of ZnS nanoparticles within the quantum-confinement regime (i.e., quantum dot dimensions).

Thus, it was possible to estimate the nanoparticle size using Brus’ equation model (eq 1),^21,44^ considering the formation of exciton quasi-particles (e^–^/h^+^ coupled pair), with *m*_e_* = 1.71*m*_0_ and *m*_h_* = 3.04*m*_0_, the mass of a stationary electron as 9.1 × 10^–31^ kg, the dielectric permittivity of vacuum (ε_0_) 8.85 × 10^–12^ F/m, and relative dielectric permittivity of media (ε_r_) 75, at room temperature. The particle size (diameter) estimated by this method was *D* = 3.4 nm.^45,46^

1 $$E_{g,QD}=E_{g,b}+\frac{h^{2}}{8R^{2}}\left(\frac{1}{m_{e}}+\frac{1}{m_{h}}\right)-\frac{1,8e^{2}}{4\pi \varepsilon_{0}\varepsilon R}$$

Upon photoluminescence (PL) characterization, the spectra exhibit a broad emission peak centered at approximately λ = 424 nm (Figure 1C). Compared to the absorption onset, an estimated Stokes shift of 110 nm was calculated, indicating the formation of optically active ZnS QDs, with the emission often showing a red shift relative to the absorption. This effect is attributed to different pathways of the electronic transition upon light excitation, followed by charge recombination and emission. The 3D plot (Figure 1D) confirms that the most intense excitation wavelength is near 300 nm, with a corresponding emission response between 390 and 450 nm.

In the context of solar energy harvesting, the photocatalytic activity of ZnS@CMC QDs under sunlight is attributed to defect-mediated electronic states. While the primary exciton transition requires *E*_g_ = 4.04 eV, as calculated from the Tauc Plot shown in Figure 1B, structural irregularities such as sulfur/zinc vacancies and surface-bound impurities can introduce “midgap” states.^21^

Considering the samples of ZnS@CMC, the needed energy of excitation covers the active excitation from the UV–B (which means, λ ∼ 280–315 nm, and *E*_g_ ∼ 3.94–4.43 eV) to the UV-A region (which means, λ ∼ 315–400 nm, and *E*_g_ ∼ 3.10–3.94 eV).

As depicted in Figure 1D, the experimental observation of strong emissions between λ = 310 nm and λ = 325 nm of excitation confirms that these QDs can be excited by lower-energy photons, potentially extending their catalytic activity into the UV-A range of the solar spectrum.

Moreover, the essential colloidal chemical characteristics of these nanosystems were assessed using dynamic light scattering (DLS, “colloidal volume”) and zeta (or electrokinetic) potential (ZP, ζ-potential). The hydrodynamic diameter (Dh) of the colloids was 24.6 ± 2.9 nm, and the ZP measured was −50.1 ± 3.3 mV, indicating that the carboxymethylcellulose has acted as an anionic macromolecular capping ligand for stabilizing the inorganic semiconductor core of ZnS QDs (i.e., ZnS@CMC (Figure 1E)). That is of paramount importance for limiting nucleation and growth of these nanoparticles during aqueous synthesis, thereby creating ultrasmall, optically active colloidal semiconductor QDs with a core–shell supramolecular morphology.

To assess key morphological and structural features of these nanosystems, high-resolution transmission electron microscopy (HR-TEM) and associated techniques were used. TEM image (Figure 2A) showed a reasonably uniform distribution of nanoparticles, with a predominant spherical morphology, and an average diameter of 3.0 ± 0.3 nm (i.e., *r* = 1.5 nm, Figure 2C). This value is below the Bohr radius reported for ZnS nanoparticles (*a*_B_ = 2.0–2.5 nm),^21,34^ confirming the quantum confinement effect and supporting the UV–vis findings discussed in the previous section. Additionally, in the HR-TEM image (inset Figure 2A), it was possible to observe electron diffraction patterns associated with the interplanar distance *d* = 0.31 ± 0.07 nm corresponding to the plane (1 1 1) of the cubic structure of ZnS (JCPDS 80-0020).

**Figure 2:**
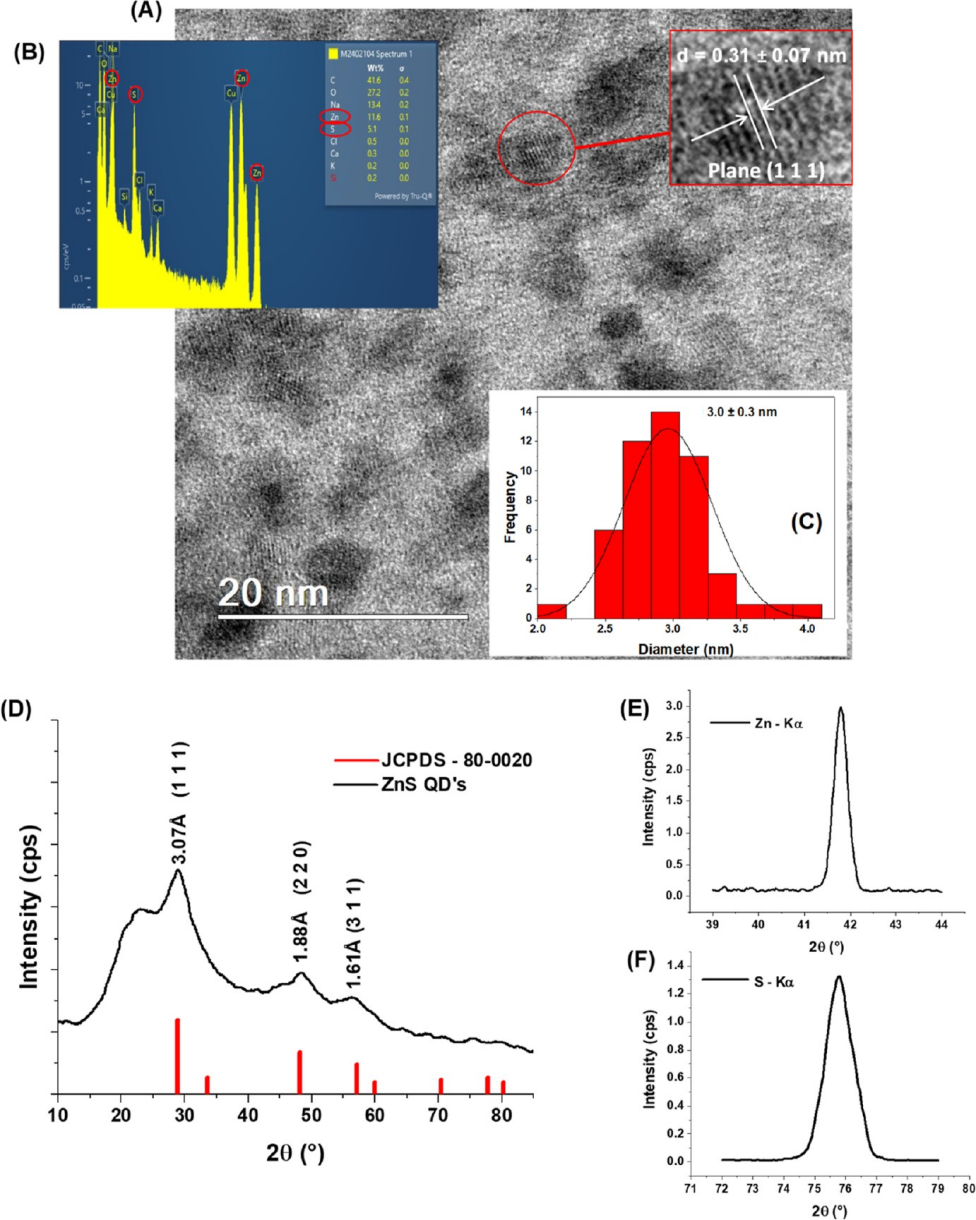
(A) TEM and HRTEM (inset) of ZnS quantum dots. (B) EDS spectrum. (C) Histogram of the sizes of quantum dots. (D) XRD of the ZnS QDs. XRF spectra of (E) Zn and (F) S regions.

The XRD (Figure 2D) patterns typically showed the presence of CMC associated with a broad band between 2θ = 15°–35°, as well as the nanocrystalline cubic structure of ZnS with three principal planes (1 1 1) with an interplanar distance of 3.07 Å, the (2 2 0) with 1.88 Å, and the (3 1 1) with 1.61 Å.^47,48^ As expected, due to the huge surface-to-volume ratio of these ZnS QDs, the XRD patterns generally show much broader bands, inferior resolution, and lower diffraction intensity.

To confirm the chemical analysis, based on the precursors and reagents used in the synthesis, EDS (energy-dispersive X-ray spectroscopy) and XRF (X-ray fluorescence) analyses were performed. As expected, EDS (Figure 2B) showed the presence of zinc and sulfur elements in the sample. Also, sodium, copper, carbon, and oxygen were detected. As expected, the detection of sodium is attributable to the use of a salt precursor of the biopolymer (i.e., carboxymethyl cellulose sodium salt), as the stabilizing agent for QDs. Even after several washing procedures with DI water during sample preparation, the counterion, sodium, remains in solution due to strong electrostatic attraction to the high density of carboxylate groups. This trend is due to the medium pH during synthesis, at which the carboxylate groups are fully deprotonated (p*K*_a_ ∼ 4.5).

Carbon and oxygen are associated with the CMC, but C is also related to Cu, along with the holey carbon metal grid used for TEM sample preparation. XRF (Figure 2E,F) confirmed this presence by measuring peaks of Zn Kα at 2θ = 41.8°, and S Kα at 2θ = 75.8°, with a well-matched estimated ratio of Zn/S = 1:1.2 (±0.2).

#### 3.1.2 Characterization of In_2_S_3_ QD/CMC Nanocolloids— In_2_S_3_@CMC

Analogously to ZnS, bulk In_2_S_3_ (indium­(III) sulfide) is a semiconductor with a midbandgap typically of 2.0–2.3 eV, which absorbs visible light (yellow to blue), potentially suitable for harvesting sunlight in environmental applications.^37,38^ In addition, it is essential to note that In_2_S_3_ nanomaterials are much less toxic than other traditional heavy-metal-based semiconductor chalcogenides (e.g., CdS, PbS, CdS/CdSe), making them a greener, more eco-friendly alternative.^37,38,40^

Hence, the In_2_S_3_ quantum dots (In_2_S_3_ QDs) were synthesized via a green, aqueous colloidal process using a CMC ligand and characterized by UV–vis spectroscopy. The results showed strong absorption, with a steep rise between 250 and 350 nm and an onset near λ = 360 nm (Figure 3A). The Tauc’s plot (Figure 3B) indicated an average bandgap of 3.46 ± 0.04 eV, resulting in a remarkable blue shift of approximately 1.1 eV, compared with the reported In_2_S_3_ bulk bandgap of 2.0–2.3 eV.^15,35–38^ The estimation of the size (diameter, D) of the In_2_S_3_ QDs by the Brus equation^36,44^ was 3.0 nm, considering *m*_e_* = 0.25*m*_0_ e *m*_h_* = 0.25*m*_0_, the mass of a stationary electron as 9.1 × 10^–31^ kg, the dielectric permittivity of vacuum (ε_0_) 8.85 × 10^–12^ F/m, and relative dielectric permittivity of media (ε_r_) 11, at room temperature. So, considering the results of high blueshift and the estimated nanoparticle size much smaller than the reported Bohr radius of In_2_S_3_ (*a*_B_ < 16 nm),^36,49,50^ a strong quantum confinement effect in these nanosystems was verified.

**Figure 3:**
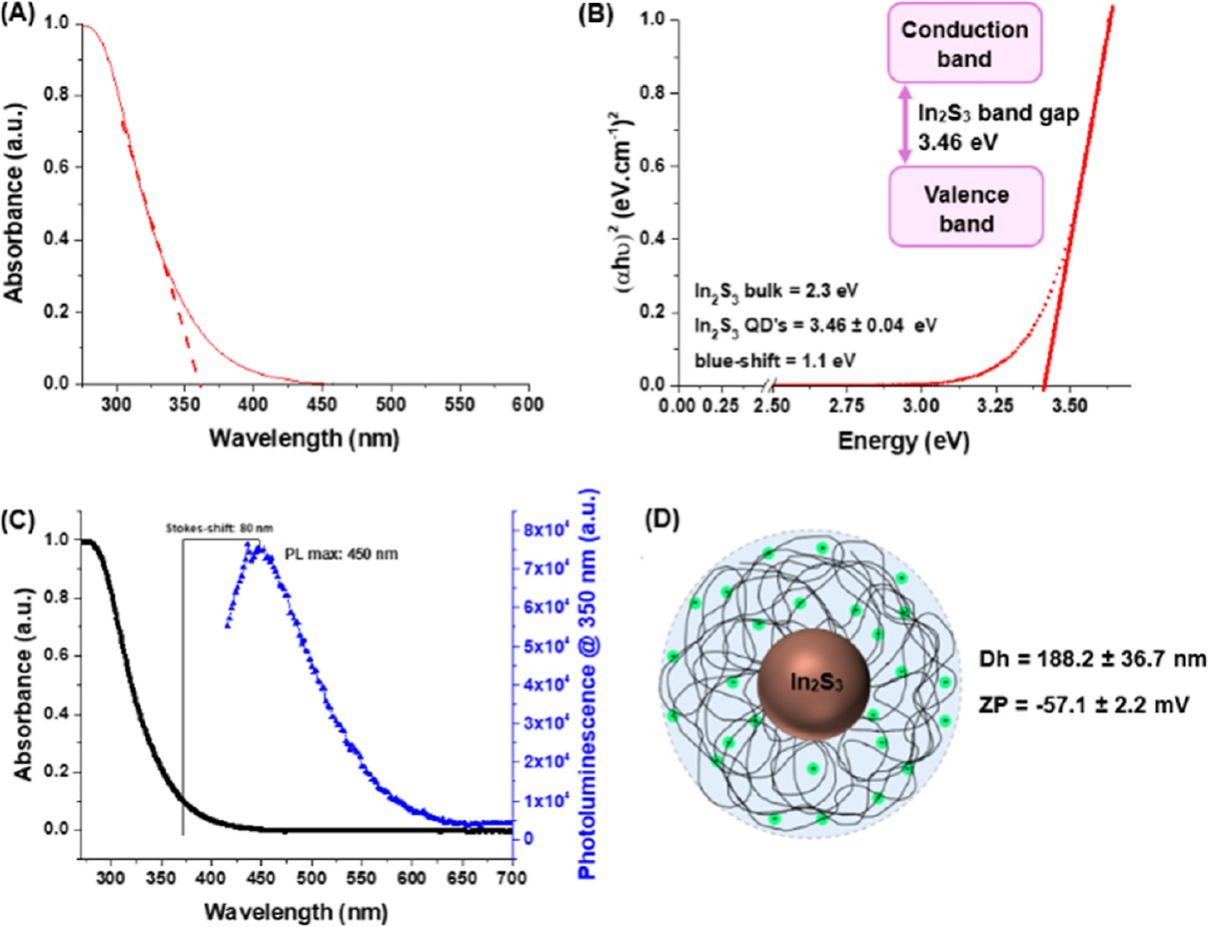
(A) UV–vis spectra for In_2_S_3_@CMC QDs synthesized. (B) Tauc plot with a schematic representation of the bandgap energy structure. (C) Absorbance and photoluminescence spectra. (D) Scheme of size and superficial charge of hybrid In_2_S_3_@CMC.

These results are consistent with the literature^36,49,50^ on similar In_2_S_3_ nanomaterials, which typically show band gaps ranging from 2.3 to 3.1 eV and are size-dependent. The photoluminescence spectra exhibited an emission peak at 450 nm, corresponding to a Stokes shift of 90 nm (Figure 3C).

The colloids were also characterized by DLS, which showed a Dh of 188.2 ± 36.7 nm, and the measured value for ZP was −57.1 ± 2.2 mV (Figure 3D).

These values, compared with those for ZnS QD colloids, indicate that the CMC chains are less agglomerated, which could be explained by the relatively lower biopolymer concentration in the media.

The TEM analysis also showed predominantly sphere-like aspects of nanoparticles with an average diameter of 2.9 ± 0.3 nm (Figure 4A,C). These values supported the UV–vis findings of a significant blueshift, confirming the formation of ultrasmall In_2_S_3_ QDs, which causes the strong quantum confinement. HRTEM image (inset in Figure 4A) demonstrated the nanocrystalline nature of particles with the presence of interference fringes with an average distance of *d* = 0.31 ± 0.0 nm that can represent the planes (2 0 6) of the beta phase of In_2_S_3_ (JCPDS 51-1160).

**Figure 4:**
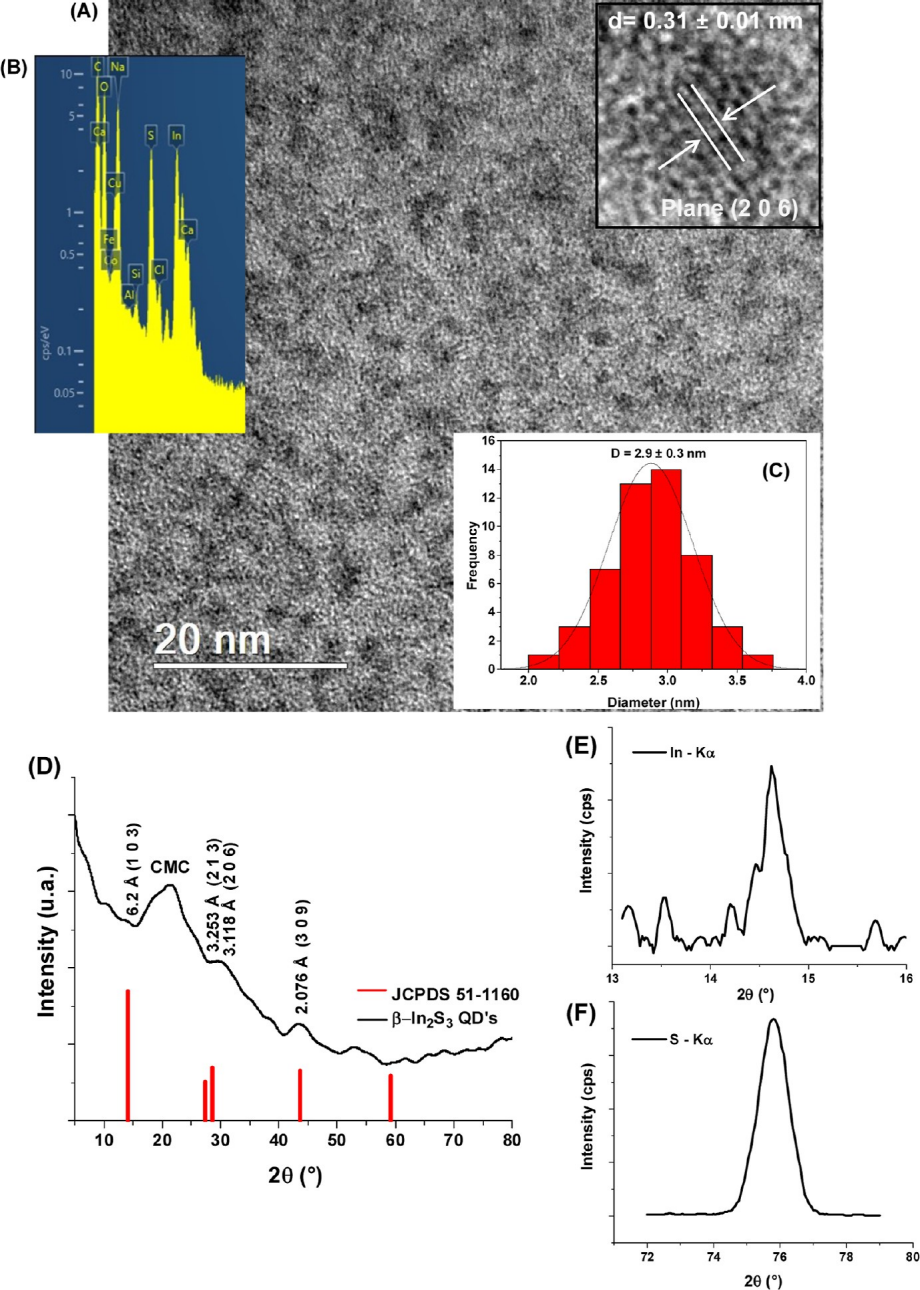
(A) TEM and HRTEM (inset) of In_2_S_3_ quantum dots. (B) EDS spectrum. (C) Histogram of size distribution. (D) XRD of the In_2_S_3_ quantum dots. XRF spectra of (E) In and (F) S regions.

The XRD analysis (Figure 4D) confirmed the β-In_2_S_3_ ^50^ as the crystalline phase, based on the prominent peaks, 2θ = 14° the plane (1 0 3) with *d*-spacing = 6.2 Å, at 2θ = 27.4° the plane (2 1 3) with *d*-spacing = 3.253 Å, at 2θ = 28.6° the plane (2 0 6) with *d*-spacing = 3.118 Å, and at 2θ = 43.5° the plane (3 0 9) with *d*-spacing = 2.076 Å. It also indicates the presence of CMC, as evidenced by the broad band between 2θ = 15° and 35°.^47,51^

The chemical analysis, as determined by EDS (Figure 4B), revealed indium and sulfur as the major elements in the samples. Similarly, XRF (Figure 4E,F) analysis confirmed their presence by the In–Kα peak at 2θ = 14.5° and the S Kα peak at 2θ = 76°, with an estimated [In/S] ratio of 2:4 (±0.4). That indicates a relative excess of sulfides to indium cations compared to the stoichiometric ratio of [In_2_S_3_] = 2:3. This trend can be attributed to the “burst-nucleation” kinetics process, which forms these nanoparticles and is far from thermodynamic equilibrium, thereby favoring an offset from the ideal stoichiometric ratio used for precursors.^52,53^

#### 3.1.3 Characterization of Zinc–Indium-Sulfide QD/CMC Nanocolloids— ZIS@CMC

As discussed in previous sections, when focusing on harvesting solar energy for photocatalytic purposes, a single nanomaterial is virtually unable to cover the entire solar spectrum. Thus, the combination of two or more components spans from the UV to the visible region of the spectrum. In this view, in addition to the ZnS and In_2_S_3_ QDs produced in this study, which represent wide- and narrow-bandgap semiconductors, respectively, their combination to form a ternary nanoalloy system was considered a viable alternative. Therefore, similar to the binary QDs, the Zn–In–S QDs were synthesized in aqueous medium stabilized by CMC, in the proportion 1:2:4 (Zn/In/S, called ZIS), based on the 50% intermediate composition of the phase diagram ZnS/In_2_S_3,_ considering these bulk semiconductor counterparts. This phase diagram is highly complex, with numerous phases that depend on composition, temperature, and other thermodynamic parameters.^54^ These ternary QDs (ZIS) were characterized, displaying a relatively broad absorption spectrum with a long tail extending into the visible range (Figure 5A). This trend is characteristic of ternary alloys, which are mainly associated with sub-band gap transitions arising from defect states.^40,55,56^ UV–vis analysis also indicated a prominent increase in absorption in the 250–350 nm range, with an onset at approximately 380 ± 2 nm. The Tauc’s plot (Figure 5B) indicated an estimated bandgap value of *E*_g_ = 3.27 ± 0.02 eV, which is, as expected, red-shifted from ZnS QDs (*E*_g_ = 4.04 eV). Thus, the ternary nanosystem ZIS@CMC supports the hypothesis of this research, serving as an intermediate-bandgap semiconductor QD that can be tuned by chemical composition, in addition to the well-known size-dependent optoelectronic properties.^23,57^

**Figure 5:**
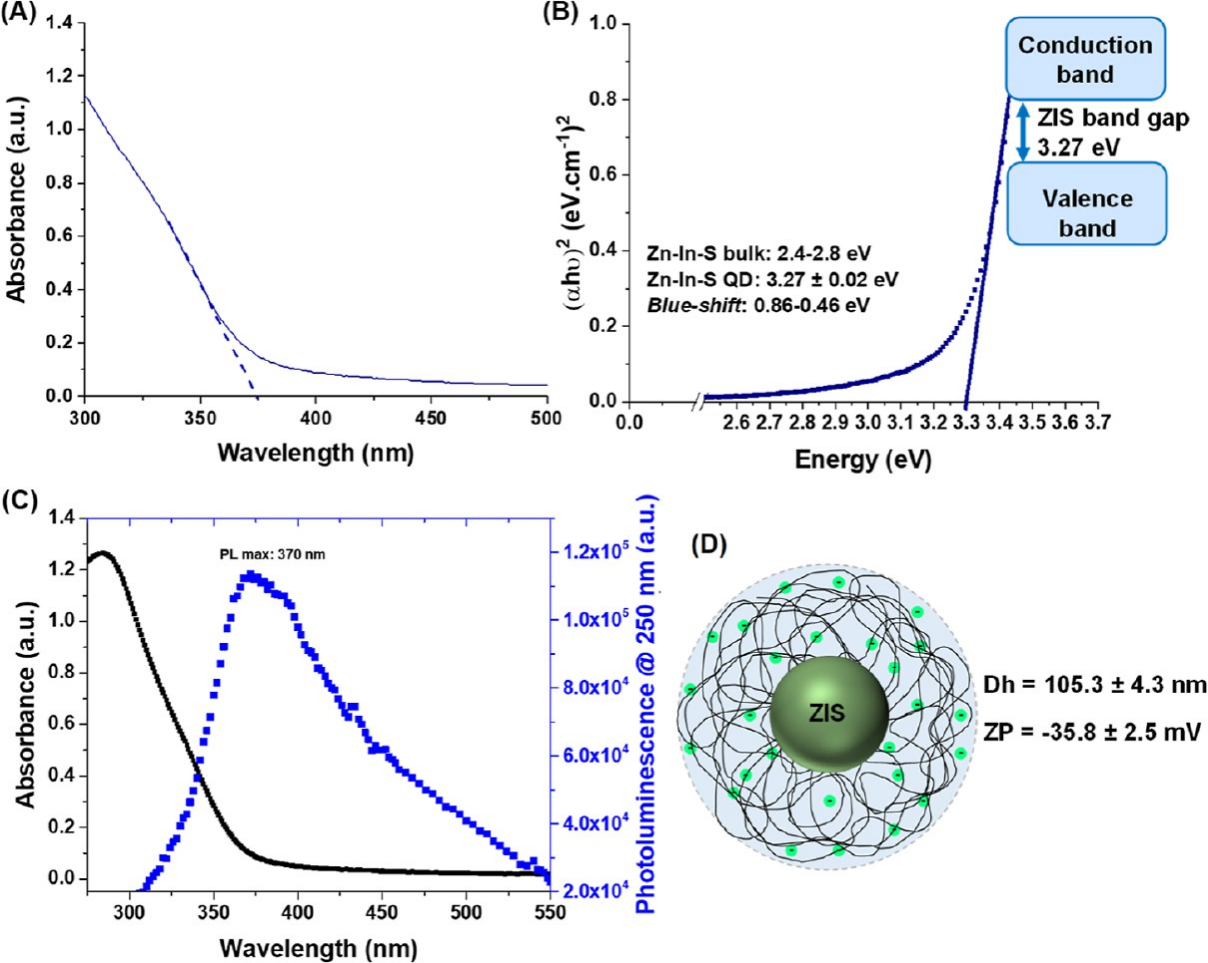
(A) UV–vis spectra of synthesized Zn–In–S@CMC QDs. (B) Tauc plot with a schematic representation of the bandgap energy structure. (C) Absorbance and photoluminescence spectra. (D) Scheme of size and superficial charge of hybrid ZIS@CMC.

Regarding the PL spectroscopy (Figure 5C), unlike binary QDs, these ternary ZIS nanosystems did not exhibit a well-defined band, most likely due to multiple recombination pathways for charge carriers (i.e., electrons and holes), consistent with the complexity of possible emissions in multinary nanomaterials.^58^

Moreover, the colloidal characterization of these hybrid nanosystems, assessed by DLS analysis, showed an average hydrodynamic size of 105.3 ± 4.3 nm and a ZP of −35.8 ± 2.5 mV (Figure 5D). So, due to the presence of CMC anionic biopolymer acting as the capping ligand, these nanocolloids were predominantly stabilized by electrostatic chemical repulsion forces in an aqueous medium.

TEM image (Figure 6A) showed predominantly relatively spherical nanoparticles, uniformly dispersed, with a fairly narrow size distribution and an estimated average diameter of 2.4 ± 0.2 nm (Figure 6C), which was significantly smaller than those of the other two types of binary quantum dots synthesized.

**Figure 6:**
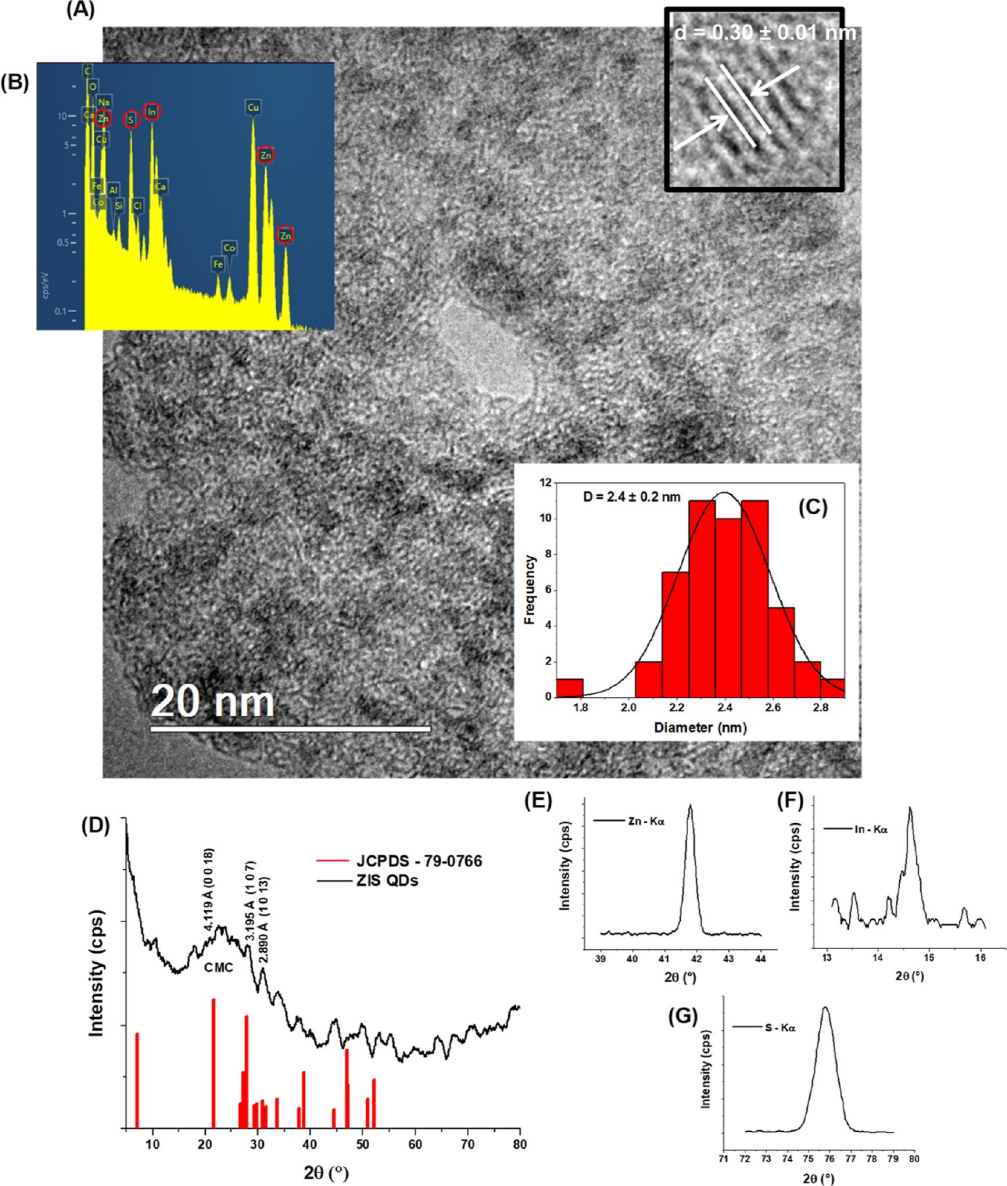
(A) TEM and HRTEM of Zn–In–S quantum dots. (B) EDS spectrum. (C) Histogram of size distribution. (D) XRD of the Zn–In–S QDs. XRF spectra of (E) Zn, (F) In, and (G) S regions.

High-resolution TEM image (inset in Figure 6A) displayed a crystalline pattern of the ZIS nanoparticles, with the estimated interplanar distance *d* = 0.29 ± 0.01 nm, compatible with the (1 0 10), (0 1 11), and (1 0 13) planes of the rhombohedral phase of ZnIn_2_S_4_ (JCPDS 79-0766). XRD analysis (Figure 6D) confirmed the presence of the (1 0 13) plane along with others, such as (0 0 18) and (1 0 7), as they are overlapped by the broad band associated with the CMC biomacromolecule.

Regarding chemical analysis, EDS (Figure 6B) confirmed the presence of the three major constituents of the ternary nanosystem, Zn, In, and S. They were also validated through XRF analysis, evidenced by the peaks of Zn Kα at 2θ = 41.8°, In Kα at 2θ = 14.6°, and S Kα at 2θ = 75.8°. In addition, the XRF results (Figure 6E–G) presented an estimated average molar ratio of these three constituents of [Zn/In/S = 1:2.6:5], which is reasonably consistent with the theoretical proportion of the precursors used in the synthesis of the ZIS ternary nanoalloy of ZnIn_2_S_4_ [Zn/In/S = 1:2:4].

In summary (Figure 7 and Table 1), for all nanophotocatalysts, a noticeable shift of the bandgap to higher energy was observed relative to their bulk semiconductor counterparts, along with visible-range absorption (Figure 7A,B). Moreover, all systems have produced tiny colloidal nanoparticles in aqueous media, with an inorganic core composed of semiconductor QD, with an average size of 2.4–3.0 nm.

**Figure 7:**
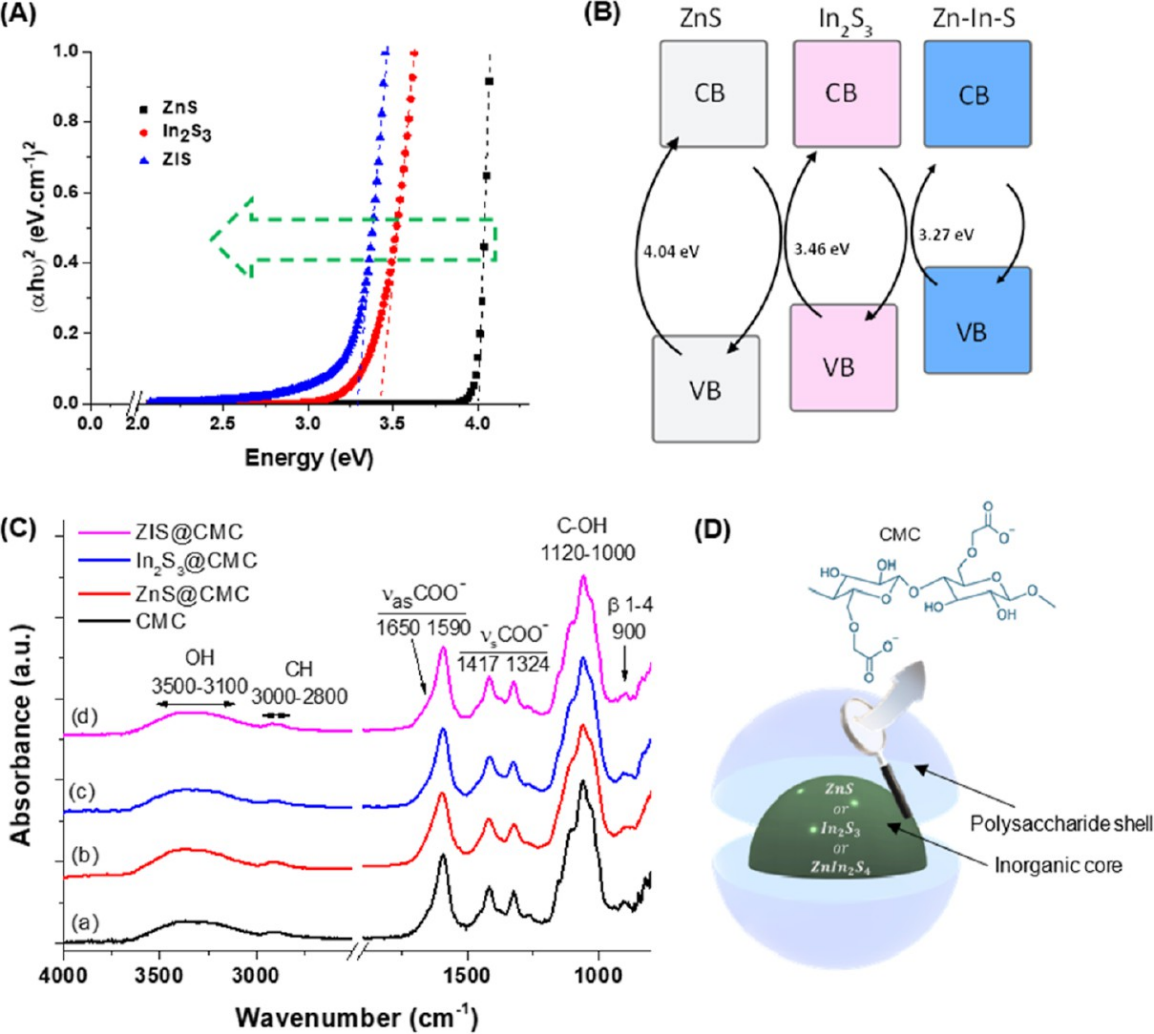
(A) Comparison of Tauc plots of ZnS@CMC, In_2_S_3_@CMC, and ZIS@CMC. (B) Schematic representation of energy band structure for the synthesized quantum dots (CB: conduction band and VB: valence band; not to scale). (C) FTIR spectra of (a) CMC, (b) ZnS@CMC, (c) In_2_S_3_@CMC, and (d) ZIS@CMC. (D) Schematic representation of the hybrid binary and ternary nanophotocatalysts.

**Table 1:** Summary of the Properties of Binary (ZnS, In_2_S_3_) and Ternary (ZIS) Quantum Dots Produced

Property	ZnS@CMC	In_2_S_3_@CMC	ZIS@CMC
Stokes-shift	110 nm	92 nm	-
Bandgap	4.04 ± 0.01 eV	3.46 ± 0.04 eV	3.27 ± 0.02 eV
Blueshift	0.42 eV	1.1 eV	0.46–0.86 eV
D (Brus’ equation)	2.9 nm	3.1 nm	-
PL max	424 nm	450 nm	370 nm
D (TEM)	3.0 ± 0.3 nm	2.9 ± 0.3 nm	2.4 ± 0.2 nm
Dh	24.6 ± 2.9 nm	188.2 ± 36.7 nm	105.3 ± 4.3 nm
ZP	–50.1 ± 3.3 mV	–57.1 ± 2.2 mV	–35.8 ± 2.5 mV

They are composed of well-dispersed and chemically stabilized by CMC macromolecular ligand, predominantly by negative repulsive electrostatic forces, as supported by FTIR analysis of CMC polymer and hybrid QDs depicted in Figure 7C and schematically represented in Figure 7D.

FTIR spectrum of CMC at pH 7.0 (Figure 7C­(a)) reveals a broad band ranging approximately from 3500 to 3100 cm^–1^, attributed to the stretching vibrations of the O–H bonds (hydroxyls/hydrogen bonds). Also, the alkyl-related stretching region (νC–H) ranges from 3000 to 2800 cm^–1^. At pH 7.0 (>p*K*_a_ of CMC), the carboxylic acids of CMC are fully deprotonated (COO^–^), and the vibrations related to carboxylates were clearly detected at 1650 and 1590 cm^–1^ (asymmetric stretching) and 1417 and 1324 cm^–1^ (symmetric stretching). In addition, the vibrational bands associated with primary and secondary alcohols (1120–1000 cm^–1^) and the vibration of glycoside bonds (β 1–4 at 900 cm^–1^) were observed.^22,31,40,47^

These findings identified the most important chemical groups of the CMC biopolymer, including the typical saccharide rings linked by ether bonds, the hydroxyl groups of cellulose, and grafted carboxymethyl groups resulting from partial substitution of –OH groups. Upon the synthesis of colloidal quantum dots, no significant changes were observed in the spectra, and the negative charge of carboxylate groups that are responsible for the colloidal stability of the quantum dot-based suspensions could be clearly seen (Figure 7C­(b–d)).

In addition, to assess and compare the chemical composition and electronic states of quantum dot nanophotocatalysts, XPS spectroscopy was employed. This surface-sensitive technique provides critical evidence for the successful formation of binary ZnS and In_2_S_3_ quantum dots, as well as their structural integration into the ternary ZIS system (Figure 8).

**Figure 8:**
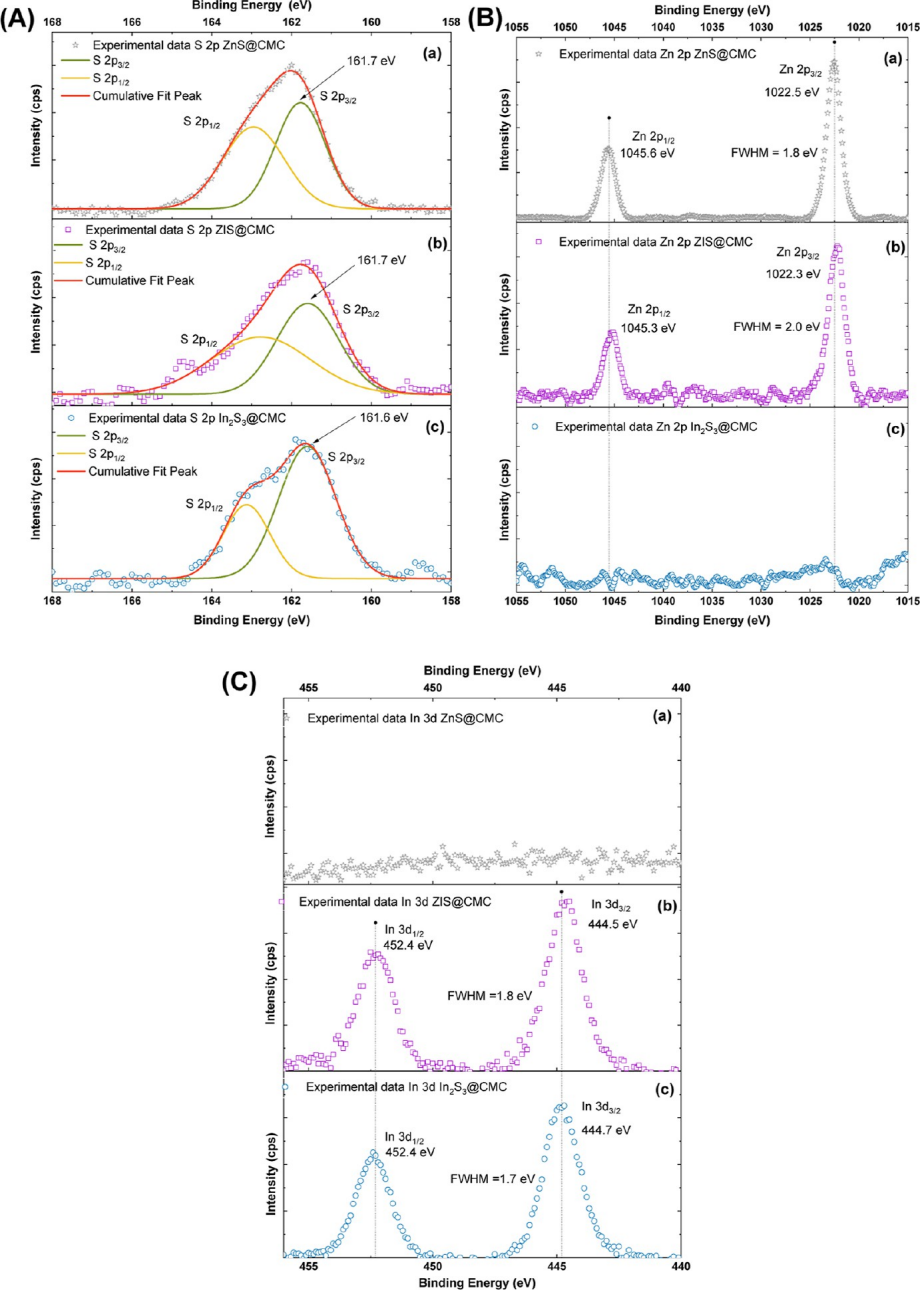
XPS narrow-spectra of (A) S 2p, (B) Zn 2p, (C) In 3d of (a) ZnS@CMC, (b) ZIS@CMC, and (c) In_3_S_2_@CMC.

The XPS narrow-spectra of S 2p (Figure 8A), Zn 2p (Figure 8B), and In 3d (Figure 8C) regions confirmed the presence of all expected elements (Zn and/or In, and S) for each QD. For ZnS, two prominent peaks at approximately 1022.5 and 1045.6 eV correspond to the Zn 2p_3/2_ and Zn 2p_1/2_ spin–orbit doublets, respectively. The consistent spin-energy separation of 23.1 ± 0.2 eV is a usual analytical fingerprint for the Zn^2+^ valence state within a sulfide lattice.^59^ Considering the S 2p spectrum of ZnS, the region shows a characteristic asymmetric peak that can be deconvoluted into the S 2p_3/2_ and S 2p_1/2_ doublet. This binding energy range is typical for S^2–^ ions in metal sulfides.^36,56,59^ Considering In_2_S_3_, similar S 2p typical sulfide signals were detected from 161 to 163 eV. The indium core-level signals appear as a well-resolved doublet at 444.7 eV, related to In 3d_5/2_, and 452.4 eV, related to In 3d_3/2_.^36^ In 3d splitting (7.7 ± 0.2 eV) also confirms the valence state of In^3+^. Zn 2p and In 3d narrow-spectra for ZIS@CMC were similar to those of ZnS and In_2_S_3_, respectively.^36,56^ S 2p spectrum further supports the formation of the ternary ZIS phase.

Compared with the binary ZnS and In_2_S_3_ samples, the ZIS@CMC displayed a subtle shift and increased full width at half-maximum (fwhm). This evolution is attributed to the formation of a complex sulfur environment in which S^2–^ anions are shared between Zn^2+^ and In^3+^ cations.

### 3.2 Nanophotocatalytic Experiments

#### 3.2.1 ZnS/MB/MO Nanosystems

The photocatalytic experiments were performed to evaluate the degradation of dyes by monitoring the maximum peak of MB (λ = 664 nm) in UV–vis spectra. The first system analyzed was ZnS QDs degrading the MB dye under UV–C (λ = 254 nm) light. There were two control samples: one without QDs (i.e., no nanocatalysts) to assess the photostability of the dye, and the other without light irradiation to assess any degradation reaction between the quantum dot and the dye (i.e., “dark”). As expected, both systems showed only a minor decrease in absorbance (<5%) in the absence of the nanocatalyst or the light source (Figure S1, Supporting Information). Then, the degradation was measured at three pH values, 3.1 (referred to as pH 3), 5.1 (pH 5), and 7.1 (pH 7), and two ratios of ZnS QD-nanocatalyst/MB-dye (23:1 and 45:1).

The photocatalysis results of ZnS QD@CMC for the degradation of MB model cationic dye are shown in Figure 9 for pH 5 and Figures S2 and S3, for pH 3 and pH 7, respectively, in the Supporting Information.

**Figure 9:**
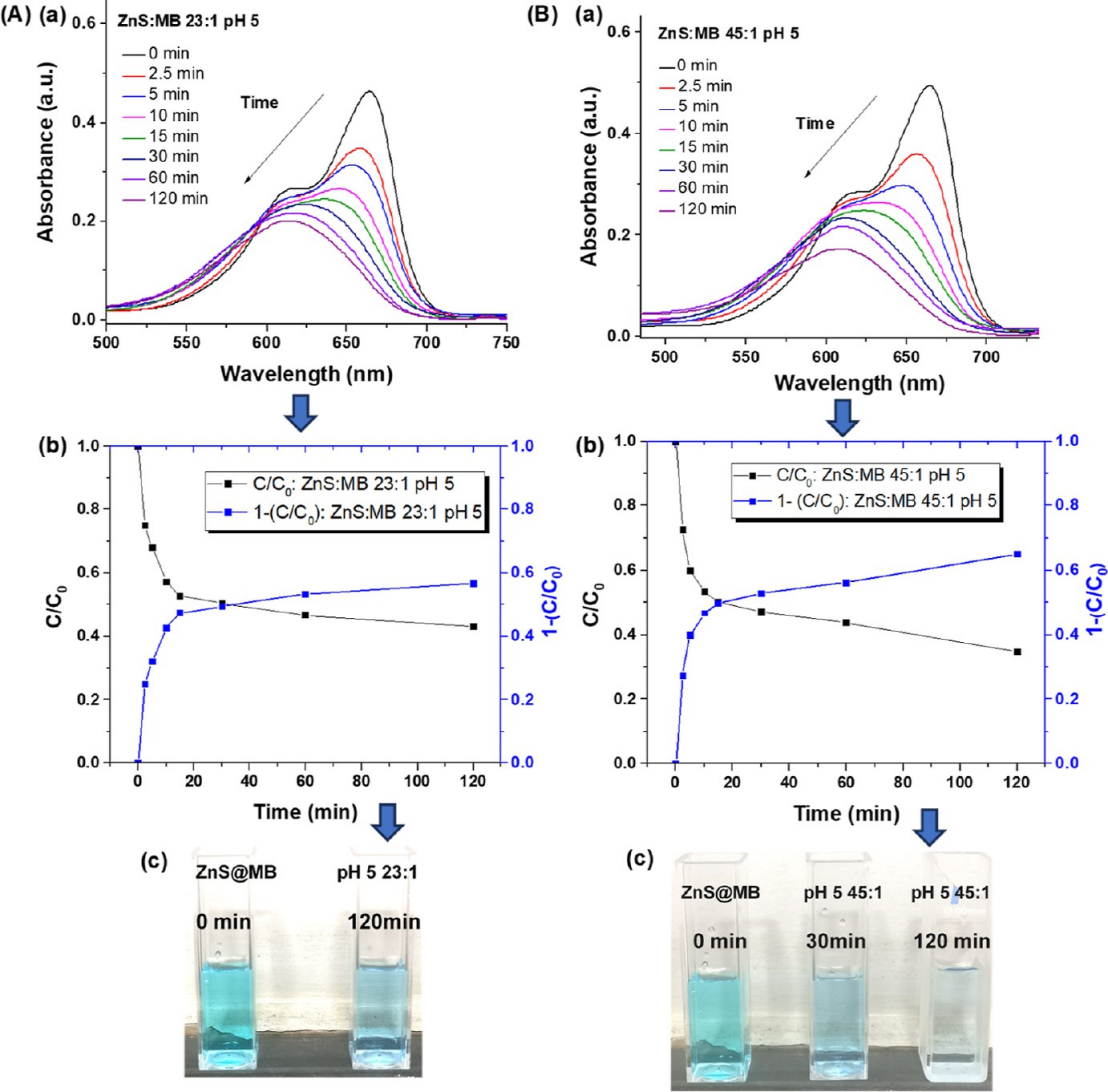
Decolorization/photodegradation assay of MB with nanophotocatalyst ZnS@CMC at pH 5: (A) ZnS/Dye [23:1] (left side) and (B) ZnS/Dye [45:1] (right side). (a) UV–vis curves with time. (b) *C*/*C*_0_ curve and degradation efficiency (1 – (*C*/*C*_0_)). (c) Digital images at *t* = 0 and after irradiation of the samples.

The results for ZnS@CMC showed the best photocatalytic efficiency at pH 5. For nanocatalyst/dye ratio of [23:1], degradation efficiency was 56.7% at 120 min with an average half-life (*t*_1/2_) of 36 min (Figure 9A­(b)) These results were significantly enhanced with a higher nanocatalyst/dye ratio of [45:1], yielding a degradation efficiency of 65.1% and an average half-life (*t*_1/2_) of 16 min, as depicted in Figure 9B­(b).

Figure 10 summarizes the effects of pH and the ZnS/dye ratio on photocatalyst behavior. These findings are consistent with an increase in degradation efficiency at higher [nanocatalyst/dye] ratios, which increases the surface area (and its atoms) available for heterogeneous catalytic reactions at the solid–liquid interface (i.e., until reactive sites are saturated).

**Figure 10:**
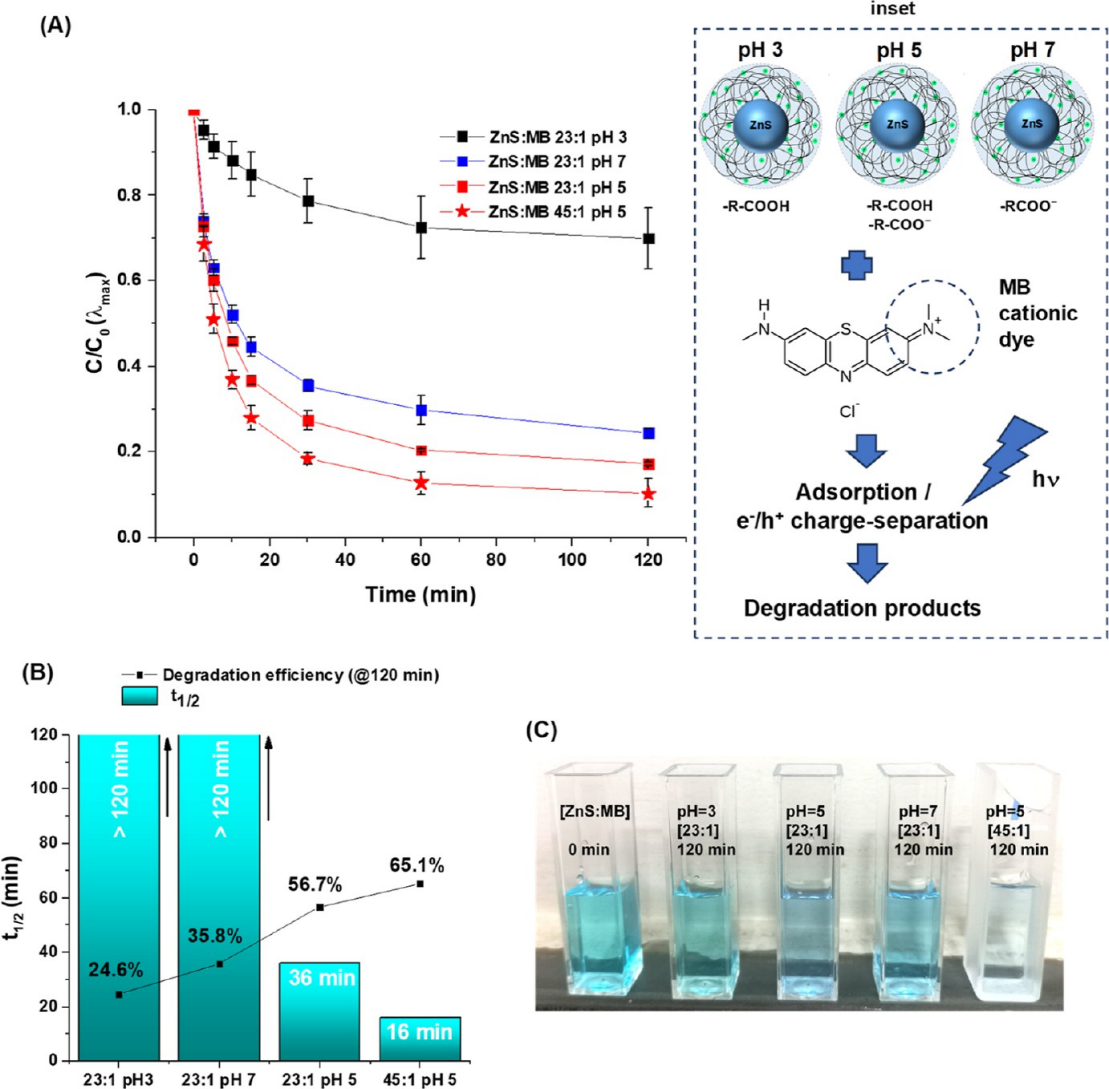
Effect of pH and Effect of Catalyst/Dye ratio: (A) Evolution of *C*/*C*_0_ of MB band (at max) with time (inset: detail of surface charges of dye and nanocatalyst and suggested mechanisms); (B) Degradation efficiency and half-life at different ZnS/MB and pH; (C) Digital Images at *t* = 0 and 120 min of irradiation of samples.

Moreover, optimal photocatalytic performance was observed at pH 5, which can be attributed to the combined effects of multiple factors, including surface charge and electrostatic interactions at the solid–liquid interface. Under mildly acidic conditions (i.e., pH 5.1 ± 0.2), the carboxylic and carboxylate groups (RCOOH/RCOO^–^) of the CMC ligand coexist at the surface of the core–shell nanostructures.

This specific ionization state creates an optimal balance that strongly favors the attraction-driven adsorption of the cationic methylene blue (MB) molecule at the catalyst interface while simultaneously promoting the pivotal charge-separation (e^–^/h^+^) redox process upon UV irradiation, promoting enhanced degradation at the interfaces.^22^ The pathways of photocatalysis were more in-depth addressed, discussed, and presented in Section 3.2.3.

Moreover, the kinetics were preliminarily evaluated using two mathematical models for the degradation of organic dyes by photocatalysis: pseudo-first order (eq 2) and pseudo-second order (eq 3).^20,21^

2 $$\ln\left(C_{0}/C_{t}\right)=k_{1}t$$

3 $$1/C_{t}=1/C_{0}+k_{2}t$$

where: where *C*_0_ and *C*_*t*_ are the concentrations of MB dye (mg/L) in the QD aqueous solution at time *t* = 0 and at a time “*t*” of UV irradiation, respectively; and *k*_1_ is the pseudo-first-order (PFO) rate constant (1/min), *k*_2_ is the pseudo-second-order (PSO) rate constant (L/mg min), and *t* is the time (min).

These models represent degradation that is predominantly limited by physical and chemical adsorption, respectively, with the PFO model being the most frequently reported.^20,21^ In this regard, all these QD@CMC nanosystems exhibited the best fit to the PSO model, as evidenced by the comparative fitting curves for both models and by the *R* ^2^ values presented in Figure 11 and Table S1 (see summary of *R* ^2^ values, Supporting Information).

**Figure 11:**
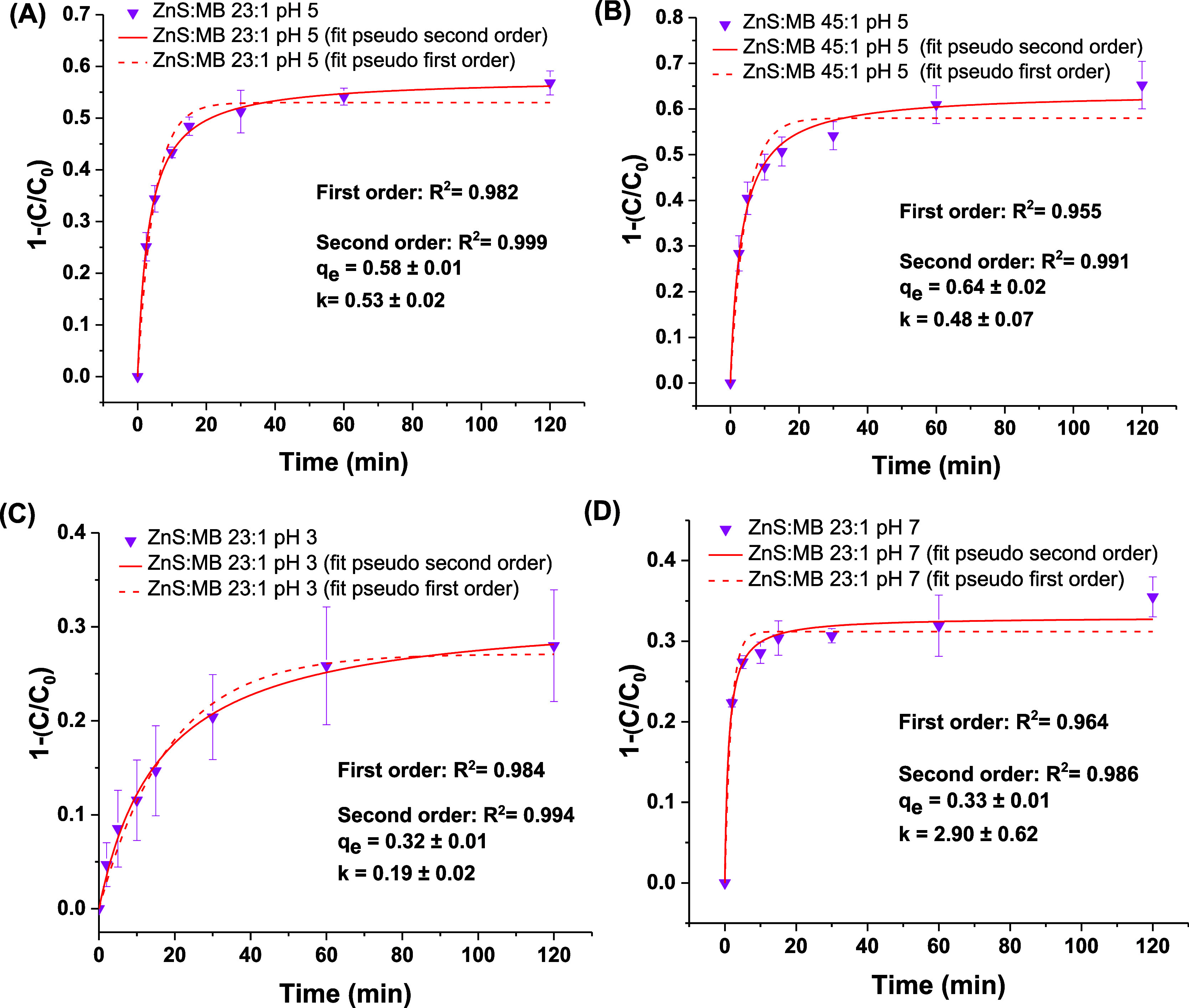
1 – (*C*/*C*_0_) regression of kinetic models of methylene blue degradation: (A) ZnS/MB [23:1] at pH 5; (B) ZnS/MB [45:1] at pH 5; (C) ZnS/MB [23:1] at pH 3; and (D) ZnS/MB [23:1] at pH 7.

Furthermore, to assess the potential application of ZnS-QD nanocatalysts for degrading anionic dye pollutants in aqueous media, methyl orange (MO), a common anionic azo dye highly toxic to ecosystems and biological environments,^60^ was also evaluated. It was tested under similar pH conditions to MB (i.e., pH 3, 5, and 7) and a nanophotocatalyst/dye ratio of [23:1] (Figures 12, and S4 (controls), and Figure S5, Supporting Information).

**Figure 12:**
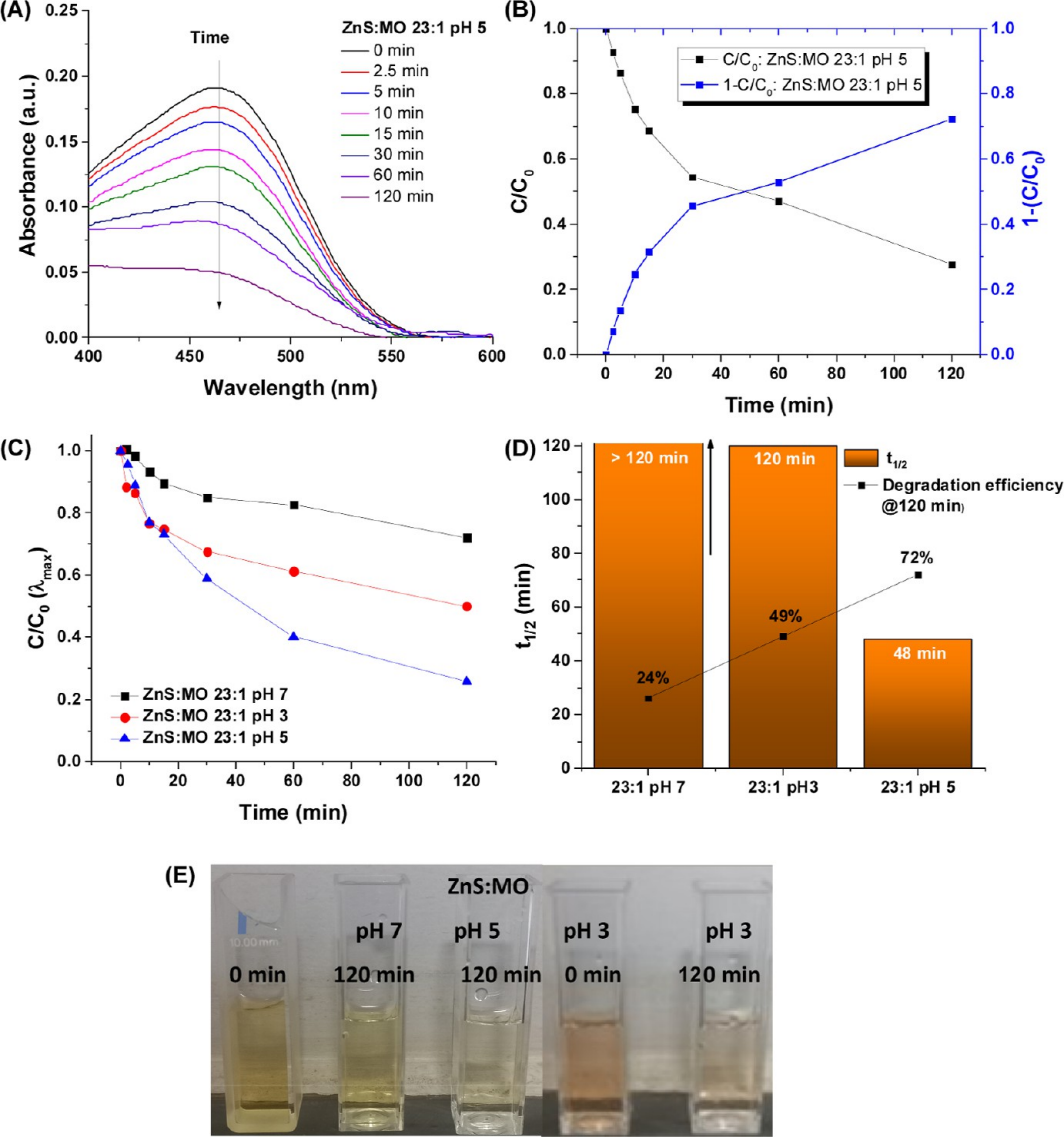
Decolorization/photodegradation assay of MO with nanophotocatalyst ZnS@CMC: ZnS/Dye [23:1] at pH 5. (A) UV–vis curves with time. (B) *C*/*C*_0_ curve and degradation efficiency (1 – (*C*/*C*_0_)). Effect of pH and effect of catalyst dye: (C) Evolution of *C*/*C*_0_ of MO band with time; (D) Degradation efficiency and half-life at different ZnS/MO and pHs; (E) Digital Images at t = 0 and 120 min of irradiation of samples.

For ZnS/MO nanosystems, equivalent characteristics were observed to those in the ZnS/MB system, where pH 5 also presented the best performance for degrading the anionic dye at 120 min of UV–C irradiation (Figures 12 and S5, Supporting Information). Nonetheless, when comparing two systems at pH 5 (MO × MB), MO showed higher degradation efficiency (72.3% vs 56.7%) after 120 min. However, the half-life (*t*_1/2_) of 48 min measured for MO nanosystems was also higher than that of the MB (*t*_1/2_ = 36 min) analog under the same experimental conditions, indicating that MO exhibits relatively slower photodegradation kinetics.

This behavior was confirmed by comparing the PSO rate constants (Figure 11 for MB and Figure 13 for MO), as MO degradation also followed an equivalent kinetic model (Table S1, Supporting Information).

**Figure 13:**
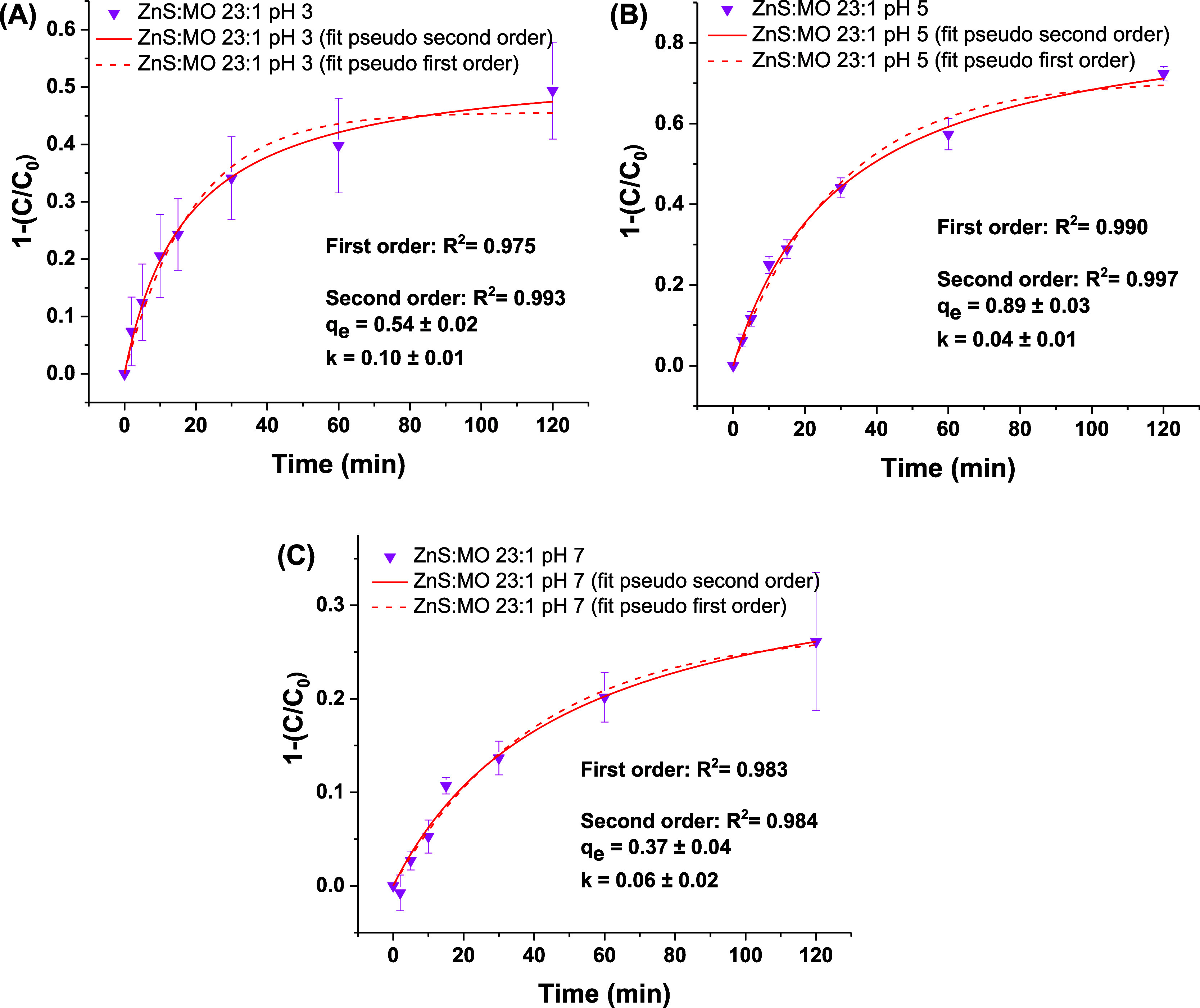
Kinetic regression models of degradation of ZnS/MO 23:1 ratio at (A) pH 3, (B) pH 5, and (C) pH 7.

These results demonstrated a superior fit between the experimental data and the pseudo-second-order (PSO) kinetic model for both MB and MO, indicating that degradation is primarily governed by chemisorption rather than by purely physical adsorption or diffusion.^20,21^

According to the literature,^20,21^ although PSO is not the most commonly used kinetic model for nanophotocatalysis, this behavior is not unexpected for the hybrid nanophotocatalyst developed in this study, which comprises a QD core and a polymer capping ligand. In fact, it can be attributed to the binding involving specific valence forces and electron–exchange interactions between the target molecules and the abundant functional groups present in the CMC. Such interactions are essential for subsequent interfacial charge transfer at the quantum dot surface. While diffusion from the bulk solution to the polymer shell might be rapid, which would favor PFO kinetics early on, the actual orientation, 3D conformation, binding, and subsequent catalytic activation of the molecule at the polymer-QD interface are complex, time-consuming processes.^20^

Regarding photodegradation responses, different results were initially expected for MO compared to MB due to repulsive electrostatic forces between the negatively charged species, CMC and MO, at the solid–liquid colloidal interfaces. That is distinct from MB, a cationic molecule that is attracted to the negatively charged CMC functional groups, thereby favoring the adsorption of the dye onto the nanocolloids and, consequently, promoting enhanced degradation at the interfaces.^22^

However, as a multifactorial and complex dynamic process co-occurring at the colloidal solid–liquid interfaces, these findings were interpreted as the overall balance of two major contributions: (a) the adsorption of the dye at the ZnS QD surface, followed by (b) the photodegradation process caused by the electron–hole pair (e^–^/h^+^) generated upon photoexcitation (i.e., charge-separation process). Hence, MB was favored for rapid adsorption due to its positive charge, which attracted the anionic CMC shell, thereby promoting faster kinetics.

By contrast, the initial physical adsorption of the anionic MO adsorption was hindered by repulsive forces at the interface, thereby reducing the reaction kinetics rate. On the other hand, the electron–hole pair (e^–^/h^+^) generated upon photoexcitation enhanced the oxidation efficiency of the MO dye, acting as a hole-scavenging moiety (i.e., favoring charge separation of the exciton) and leading to higher degradation efficiency at 120 min^22,61^

#### 3.2.2 In_2_S_3_/MO/MB and ZIS/MB Nanosystems

To avoid redundancy with previously discussed ZnS-based nanosystems, the photocatalytic results of In_2_S_3_ binary QDs and ZIS ternary QDs tested at pH 5 were summarized in Figure 14.

**Figure 14:**
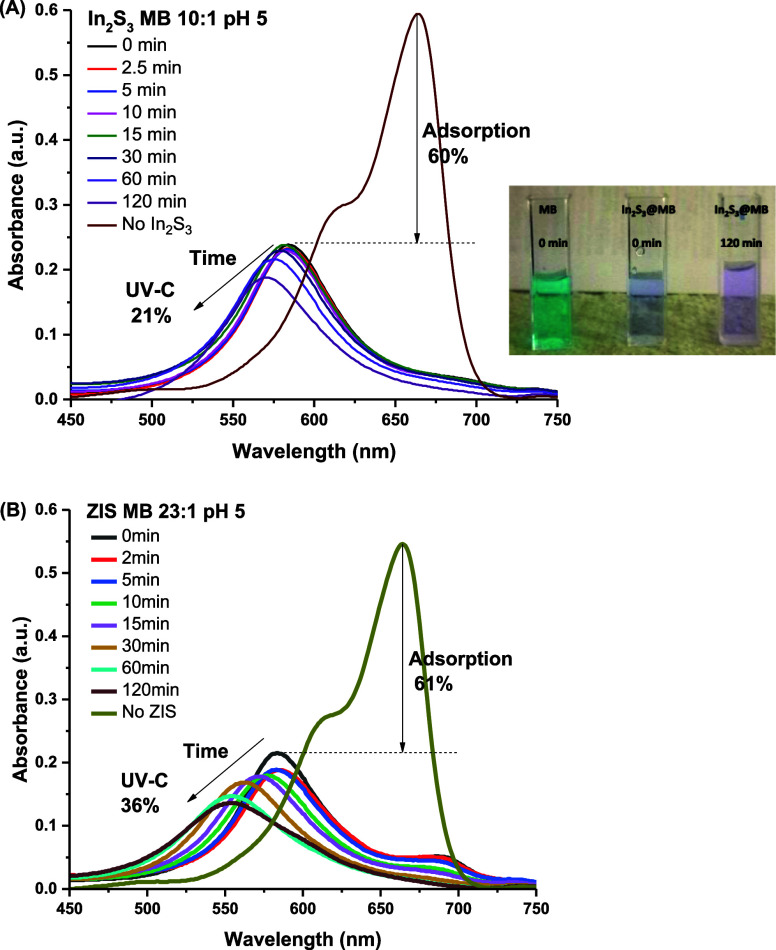
Time-dependent UV–vis spectral changes of MB associated with adsorption and photocatalysis mediated by (A) In_2_S_3_@CMC (In_2_S_3_/MB [10:1], pH 5). Inset: Digital Images at t = 0 min, before and after QD addition, and after 120 min of irradiation of samples. (B) ZIS@CMC (ZIS/MB [23:1], pH 5).

For the MB cationic dye, both QD systems (In_2_S_3_ and ZIS) showed fading of the solution color, accompanied by a reduction in the characteristic band of MB in the UV–vis spectra. Similar behavior was also observed by Ük et al. (2024) for In_2_S_3_, ZnIn_2_S_4_ QDs in solid-state matrices,^62^ which was associated with the potential of indium-based materials to adsorb dyes due to structural characteristics of In cations, where the typical valence state (In^3+^) generates Lewis acid sites that can capture and interact with organic molecules by coordinated covalent bonding.

Approximately 60% decolorization was observed for both systems upon mixing, followed by an additional 21% and 37% decolorization upon irradiation with a 254 nm UV–C lamp (after 120 min) for In_2_S_3_ and ZIS, respectively. The combined adsorption and photocatalysis resulted in 68% and 75% total MB dye removal by binary and ternary QDs, respectively, and a 20% reduction in UV–vis intensity.

It was also observed that the blue shift of the peak is associated with a visual color change of the solution from blue to violet.

According to the literature,^16^ this change can be attributed to the demethylation process of the dye, in which a bond is broken, and methyl groups are released, yielding coproducts such as Azure A, Azure B, Azure C, or thionine. Due to its violet color and a peak at approximately 560 nm, thionine was the likely compound formed after 120 min.^16^

Regarding the MO anionic dye, it was not detected to be adsorbed or removed upon contact with In-containing QDs, as reported in the literature.^62^ Upon irradiation of UV–C 254 nm light, there was a slight decrease in MO absorbance (decolorization/degradation) of about 30% (Figure S6, Supporting Information).

The observed photocatalytic activity of both ZnS@CMC and ZIS@CMC, despite their different calculated band gaps of 4.04 and 3.27 eV, respectively, can be attributed to a defect-mediated excitation mechanism. In these quantum-confined systems, the high density of surface defects and sulfur vacancies can create localized intermediate states within the forbidden band. These substates enable absorption of lower-energy radiation via sub-bandgap transitions, thereby facilitating electron–hole (e^–^/h^+^) separation without requiring high-frequency UV–C photons.^63^ While ZnS primarily relies on discrete defect levels to exhibit solar-harvesting activity, the ternary ZIS system benefits from a hybridized electronic structure.^64^ This “defect-engineering” inherent to the aqueous colloidal synthesis ensures that both nanophotocatalysts remain active under environmentally relevant light conditions, producing the necessary ROS for the mineralization of MB and MO dyes.

#### 3.2.3 Spectroscopic Insights into the Oxidative Transformation of MB at the Polysaccharide-capped Interface and Photocatalytic Efficiency

As previously reported,^16^ the primary degradation pathway of MB involves a stepwise N-demethylation process in which the radicals attack the dimethylamine groups, sequentially removing methyl groups. This process breaks the molecular conjugation, leading to gradual decolorization (i.e., color fading), followed by ring cleavage and complete mineralization.^16^ The loss of methylene groups results in color changes from the initial, blue-colored, MB molecule to Azure B and Azure A. These are the most usual intermediates formed upon loss of one or two methyl groups, respectively. The fully demethylated intermediate, thionine, is purple and is shown in the inset of Figure 14A.

Additionally, the products of complete MB oxidation include more oxidized species, such as carbon dioxide, inorganic ions, and sulfates, according to published reports.^16,63^ The photocatalytic activity of ZnS@CMC, In_2_S_3_@CMC, or ZIS@CMC can initiate the aforementioned MB degradation pathway.

According to the literature,^7,64–66^ such a process can be initiated by the photogeneration of e^–^/h^+^ pairs, in which, upon photoexcitation with sufficient energy, the induced internal electric field facilitates their spatial separation and subsequent migration to the surface.

Under such conditions, surface-bound active sites catalyze the degradation process by reacting with surface-adsorbed O_2_ to yield ^•^O_2_^–^ radicals. In the meantime, the positive holes in the valence band interact with H_2_O, primarily generating ^•^OH radicals. The combined effect of these reactive oxygen species (ROS) can lead to the oxidative degradation of the dye, transforming it into stable, mineralized, nontoxic end products, which has intesively been intensively researched.^22,27–30,64,67^

Conversely, if a rapid recombination of the photogenerated electron–hole pairs occurs, it will hinder the photocatalytic degradation of dyes. Thus, the charge-separation process is driven by the presence of scavenger species at the solid–liquid interface, leading the carriers to migrate to the surface of metal-sulfide QDs (Mx-Sy, binary and ternary; M = Cd, Zn, Pb, In, Cu, Sn, etc.). Some of the multiple stages comprising the photodegradation pathways of organic dyes by nanocatalysts are schematically illustrated as follows^22,68^ (Stages 1–4, eqs 4–9).

- Stage 1—Generation of coupled electron–hole pair (e_CB_^–^/h_VB_^+^, exciton) by UV irradiation of M-S (metal-sulfide) nanophotocatalysts, where the photon energy, hν_exc_ = 4.88 eV (at 254 nm) > ZnS QD *E*_g_ = 4.04 eV > In_2_S_3_ QD *E*_g_ = 3.46 eV > Zn–In–S QD *E*_g_ = 3.27 eV:

4 $$M-S\;QD+hv\left(\lambda =254\;nm\right)\to M-S\;QD\left(e_{CB}^{-}/h_{VB}^{+}\right)$$

- Stage 2— Migration of the carriers (e_CB_^–^/h_VB_^+^) to the QD surface:

5 $$M-S\;QD\left(e_{CB}^{-}+h_{VB}^{+}\right)\to M-S\;QD\left(e_{surf}^{-}/h_{surf}^{+}\right)$$

- Stage 3—Generation of reactive radicals by (e_surf_^–^/h_surf_^+^):
- (3A) Oxidation by (h_surf_^+^) of adsorbed water, generating reactive hydroxyl radicals

6 M−SQD(hsurf+)+H2O(ads)→M−SQD+O·H(ads)+H+

(3B) Oxidation by (h^+^_VB_) of adsorbed hydroxides, generating reactive hydroxyl radicals

7 M−SQD(hVB+)+OH(ads)−→M−SQD+O·H(ads)

(3C) Reduction by (e^–^_surf_) of adsorbed oxygen, generating reactive superoxide radicals

8 M−SQD(esurf−)+O2(ads)→M−SQD+O·2(ads)−

- Stage 4—Oxidation of dye pollutants by photogenerated highly reactive radicals:

9 O·2(ads)−+O·H(ads)+dye(ads)→degradedbyproducts(degradationofthepollutanttosmallermoleculeswithlesstoxicity)→H2O+CO2(mineralization)

Hence, based on comparative performance screening, the ZnS@CMC system for MB degradation at pH 5 was selected as the benchmark for in-depth validation of dye degradation. The mineralization is confirmed by a multitechnique approach comprising UV–vis and XPS spectroscopies. Both techniques bypass the need for liquid-phase separation. In contrast, chromatographic and mass spectrometry techniques (e.g., HPLC, GC–MS, and LC–MS), which are usually the gold standard for mapping specific intermediate degradation byproducts, would introduce severe interference due to the polymeric nature of the stabilizing ligand.

Because the dye intermediates are likely to be bound to the CMC and adsorbed dyes (hydrogen bonds, electrostatic interactions, hydrophilic, and hydrophobic interactions), filtering of the QDs@CMC/dye/intermediates system to obtain intermediates/byproducts for analysis could lead to false negatives and/or skewed interpretation of the degradation pathway.

Considering UV–vis signals, a simultaneous decrease in both the visible maxima, related to color, and the UV-region absorbance, related to aromaticity, was observed (Figure S7, Supporting Information), suggesting ring-opening rather than simple decolorization.^65^

Also, a detailed analysis of the UV–vis absorption profiles reveals a significant superposition in the ultraviolet region between the characteristic bands of MB and the intrinsic absorption edge of the ZnS QD photocatalyst. Specifically, while the band centered at about 290 nm,^64^ associated with the π→π* electronic transitions of the aromatic rings in the MB dye, is not eliminated, its substantial reduction in intensity provides clear evidence of the oxidative cleavage of the MB heterocyclic framework.^65^ It is essential to highlight that this decrease in intensity cannot be attributed to photodegradation or leaching of the ZnS nanostructures.

This result is corroborated by the XPS spectra of the catalyst components, shown in Figure 15A, which demonstrate that the binding energies and atomic concentrations of the Zn 2p signals remain constant, with no significant shift or broadening after the reaction, indicating that the chemical environment of the Zn remains stable as a sulfide. Therefore, they confirmed that spectral changes are exclusively due to the transformation of the organic pollutant, with the residual UV absorbance representing the baseline contribution of the chemically stable inorganic semiconductor core, i.e., acting as “true” nanophotocatalyst.

**Figure 15:**
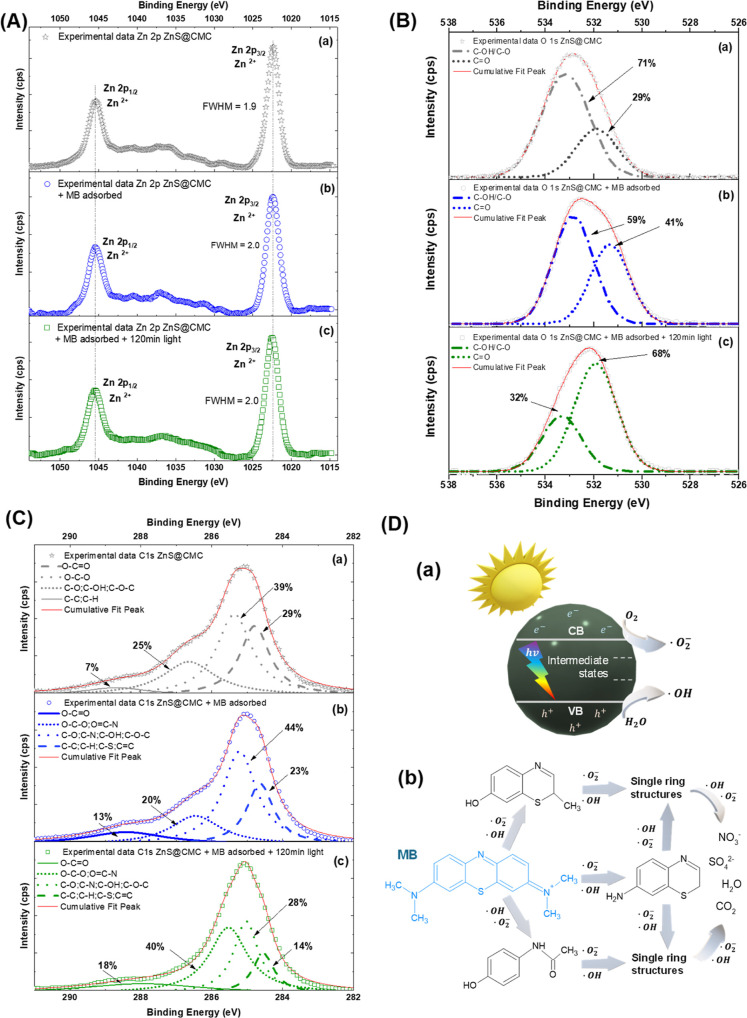
XPS spectra of (A) Zn 2p, (B) O 1s, and (C) C 1s for (a) ZnS@CMC, (b) ZnS@CMC + adsorbed MB before light exposure, and (c) ZnS@CMC + adsorbed MB after 120 min of light exposure. (D) (a) Generation of oxidative species by ZnS@CMC upon light exposure. (b) Schematic pathway of MB photodegradation by ZnS@CMC.

Moreover, as XPS is an advanced surface-sensitive technique that probes the top ∼10 nm of a sample, it is particularly effective at detecting adsorbed intermediates that remain on the catalyst surface after photocatalysis, which occurs at heterogeneous solid–liquid interfaces.^69^ Thus, aiming to validate the occurrence of MB degradation on the surface of ZnS@CMC following light exposure, XPS narrow-spectra of O 1s (Figure 15B) and C 1s (Figure 15C) were collected for bare ZnS@CMC, ZnS@CMC + MB (adsorbed) before light exposure, and ZnS@CMC + MB following 120 min of light exposure. The observed shift in the surface signals provides compelling evidence of the oxidative pathway of the photocatalytic process.

When comparing the XPS spectra of the ZnS@CMC surface from the initial adsorption phase (curves (b)) to the postdegradation state (curves (c)), a distinct increment in the ratio of oxidized carbon species (such as C–O–, C=O, and O–C=O) relative to the total oxygen or carbon content was observed (measured as % of area of each peak contribution). This evolution in the C 1s and O 1s spectra suggests that MB molecules are attacked by photogenerated reactive oxygen species, leading to cleavage of the heterocyclic framework and the formation of oxygenated organic intermediates.^64–66,70^

The accumulation of these carbonyl and carboxyl residues at the nanocatalyst interface, surrounded by the CMC polysaccharide backbone, indicates a progressive mineralization process.

While the inorganic core remains structurally undamaged—as evidenced by the constant Zn signals—the shifting carbon-to-oxygen environment confirms that the organic dye is being effectively transformed into more oxidized, and ultimately nontoxic, chemical species. The schematic pathway of MB photodegradation by ZnS@CMC is summarized in Figure 15D.

#### 3.2.4 Comparison with Literature and Future Directions

Regarding photocatalytic efficiency, although the ZnS@CMC system achieved up to 65% degradation of MB and 72% of MO within 120 min, which falls within the performance range of other Zn/In/S-containing QD-based systems,^63^ it is imperative to situate this performance within the broader context of semiconductor-mediated photocatalysis. According to recently emerged accounts, addressing inherent shortcomings, such as substantial recombination of photogenerated charge carriers, QDs for photocatalysis applications require, besides the stabilized surface area, innovative strategies, such as the development of organic–inorganic heterojunctions and defect engineering (i.e., the intentional introduction of vacancies and other defects in the semiconductor structure).^63,64^ Further, there is an increasing emphasis on pursuing sustainable production routes and elucidating the underlying charge-transfer processes and radical-involved mechanisms, which currently garners more research effort than merely pursuing higher dye conversion efficiencies.^63,64^

In addition, for the QDs developed in this work, ZnS@CMC, In_2_S_3_@CMC, and ZIS@CMC, the presence of the organic layer introduces complex interfacial dynamics that significantly modulate photocatalytic efficiency, as the supramolecular structure in aqueous media involves hydrogen bonding, electrostatic interactions, and hydrophilic–hydrophobic interactions between the medium and the nanoparticle surface.^22,59^ Strong electrostatic interactions between the ionized functional groups (e.g., carboxyl) and/or hydroxyl groups of CMC and the cationic MB molecules can promote the “preconcentration” of the dye near the inorganic surface, effectively increasing the local collision frequency with the generated radicals. Conversely, the chemical groups inherent to the ligand backbone may act as “parasitic traps”, in which specific moieties can facilitate charge recombination by capturing photogenerated holes or electrons before they reach the surface-active sites.^21,31,59^

Additionally, dense polymeric regions can repel similarly adjacent charged species, whereas steric hindrance may reduce the diffusion rate of the dye toward the reactive interface. Therefore, the overall net degradation process is governed not only by nanoparticle size and/or the amount of active surface states, but also by a delicate balance between the ability of the polymer to recruit dye molecules via supramolecular affinity and its potential to act as a barrier to charge transport or a site for nonradiative recombination.

To directly contextualize the performance of the proposed CMC-stabilized aqueous nanosystems within the broader landscape of environmental nanotechnology, Table 2 summarizes the organic dye photodegradation efficiencies and basic catalyst compositions of quantum dot-based photocatalysts reported in the recent literature. However, it should be noted that the synthesis methods, parameters, compositions, (nano)­structures, and processes used to produce these nanomaterials are distinct and therefore cannot be straightforwardly compared.

**Table 2:** Summary of Organic Dyes Photodegradation Efficiencies and Nanocatalyst Compositions for QD-Based Nanosystems^a^

type of nanostructure	type of pollutant	efficiency of degradation (%)	ref
ZnS/Chitosan	MB, MO	87–MB	^21^
		68–MO	
ZnS/poly-l-lysine—iron oxide/CMC	MB	34	^22^
ZnS/Chitosan Nanocomposite	CV, AR-I	93.4–CV	^30^
		90.7–AR-I	
β-In_2_S_3_ QDs embedded in Nafion matrix	Rhodamine-6G	95	^50^
In_2_S_3_ in solid-state oleic acid matrices	MB, MV, RB, MO, NF	98.9–MB	^62^
		95.0–MV	
		66.5–RB	
		42.1–MO	
		38.4–NF	
ZnIn_2_S_4_ in solid-state oleic acid matrices		56.1–MB	
		79.7–MV	
		59.5–RB	
		8.6–MO	
		8.6–NF	
Cu/ZnIn_2_S_4_ in solid-state oleic acid matrices		51.9–MB	
		93.7–MV	
		82.1–RB	
		7.5–MO	
		12.1–NF	
Biogenic TiO_2_ Nanoparticles	MB, RB	99.4–MB	^65^
		99.3–RB	
NiS–ZnS/Polyvinylpyrrolidone	MB, Rose Bengal	96.9–MB	^67^
		97.1–Rose Bengal	
BiFeO_3_–Black TiO_2_ Composite	MB	97	^71^
CMC-stabilized QDs	MB, MO	65–MB	This Work
		72–MO	

a Abbreviations: Rhodamine B (RB), Crystal Violet (CV), Acid Red-I (AR-I); Sodium fluorescein (NF), Methyl violet 2B (MV).

While the photodegradation efficiencies achieved by the nanocatalysts developed in this work (up to 65% for MB and 72% for MO) are relatively moderate when compared to highly engineered QDs or traditional powder-based heavy-metal catalysts that can reach near-complete removal,^63–66,71,72^ this aqueous colloidal nanosystem offers significant advantages from a sustainable engineering perspective. It integrates an environmental nanotechnology strategy with a “biosafe by design” approach. The integration of nanosystems with biobased polymers (i.e., natural or semiprocessed biopolymers) ensures a low-toxicity, biodegradable framework that aligns with sustainable chemistry principles. Furthermore, the supramolecular architecture—arising from the polysaccharide-based shell—provides exceptional colloidal stability. Water-dispersible systems circumvent the energy-intensive drying processes required for traditional powder-based catalysts, integrating more readily into existing industrial wastewater treatment infrastructures. Hence, the transition from powder systems to biobased aqueous colloidal systems can confer strategic advantages in the context of modern green chemistry and industrial scalability.

From a critical perspective, despite these very promising results, further studies are required to optimize the kinetics and efficiency, and to address the charge-separation process of these novel nanophotocatalysts in the visible range of the solar spectrum. Also, multicycle stability assays, catalyst recovery methods, and performance evaluations in real wastewater matrices are critical. These next steps are to bridge the gap between this green-synthesis proof-of-concept and the viable pilot-scale stage for actual applications. Moreover, biopolymers such as polysaccharides, despite the numerous advantages previously described, as they are sustainable, renewable, and naturally sourced, can lead to variations in composition and structure, posing a challenge in large-scale industrial applications for water treatment technologies.

In summary, the envisioned practical applications of the synthesized hybrid nanocatalysts are schematically depicted in Figure 16. Ideally, by harnessing natural sunlight to drive photocatalysis, these polysaccharide-capped QDs could help restore environmental equilibrium by mitigating the hazardous effects of dye pollutants (i.e., from Figure 16A–C).

**Figure 16:**
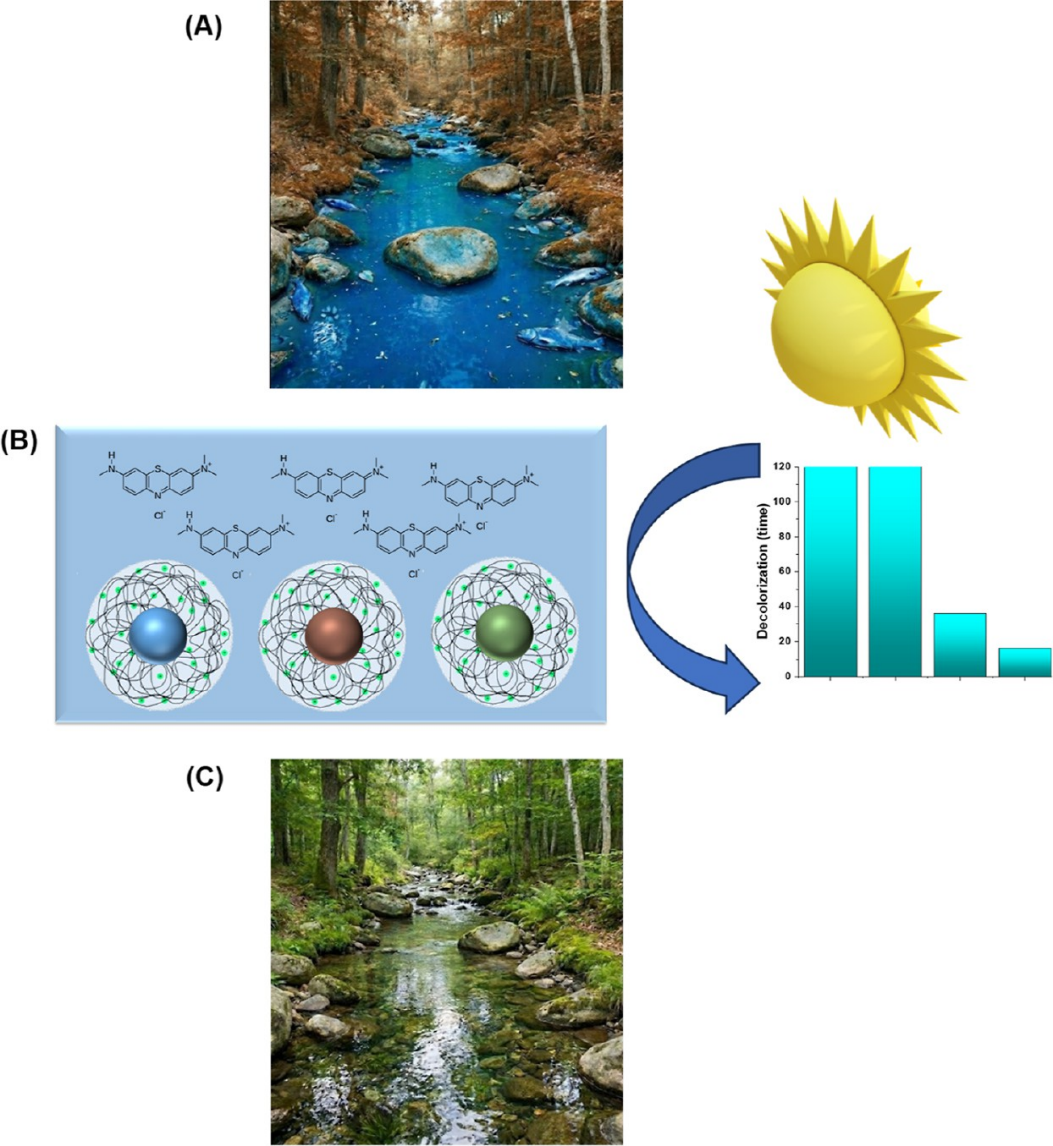
Schematic representation of the projected application of these nanohybrids for cleaning dye spill accidents in water streams and animals. (A) AI simulation of contamination with a blue dye spill affecting fish and severe environmental impact. (B) Representation drawing of the quantum-dot/biopolymer nanohybrids (QD@CMC) promoting the degradation of dye molecules through sunlight-mediated photocatalysis (C). AI simulated after the full remediation from the hazardous impact (blue color removed from the image background in (A)), ideally restoring the original environmental equilibrium.

It can be projected that, in a broader perspective, beyond water remediation, coupling QD-based photocatalysts with solar-driven or hybrid renewable energy systems could provide sustainable, decentralized solutions for clean water production, particularly in remote or resource-limited areas. In this scenario, over the following decades, the role of nanosized photocatalysts is poised to grow significantly, provided that current challenges related to nanosafety, legislation and regulation, and large-scale production technology are successfully addressed by future studies and advancements.

## 4 Conclusion

This study demonstrates the successful synthesis of eco-friendly colloidal binary (ZnS and In_2_S_3_) and ternary (Zn–In–S, ZIS) quantum dots, chemically stabilized with carboxymethyl cellulose (CMC), via a green aqueous route. Extensive structural, morphological, and optical characterization confirmed their crystalline nature, nanosize dimensions (∼2–3 nm), and tunable band gaps within the range of 4.04–3.27 eV, which were demonstrated to be dependent on the composition of the inorganic core. When applied as nanophotocatalysts for the degradation of model organic pollutants, cationic (methylene blue) and anionic (methyl orange) dyes, these nanosystems exhibited removal efficiencies of 30–70%, with optimal activity observed at pH 5 under 2 h irradiation. The degradation kinetics were best described by a pseudo-second-order model, consistent with a chemisorption-driven process. Despite similar mathematical models, the cationic MB dye favored early stage adsorption onto the negatively charged ZnS@CMC compared with the anionic MO dye. On the other hand, MO has demonstrated a relatively higher degradation efficiency, credited to its hole-scavenging characteristic, which favors the oxidation of the organic dye. These findings highlight the potential of CMC-stabilized QDs as promising, sustainable nanophotocatalysts for advanced water treatment and dye remediation, offering an environmentally benign, facile pathway to mitigate organic dye pollution.

## Supplementary Material



## Notes

Figure S1—Controls of ZnS/MB catalysis assays; Figure S2—Decolorization/photodegradation assay of ZnS/MB [23:1] at pH 3; Figure S3—Decolorization/photodegradation assay of ZnS/MB [23:1] at pH 7; Figure S4—Controls of ZnS/MO catalysis assays; Figure S5—Decolorization/photodegradation assay of ZnS/MO [23:1] at pH 3 and pH 7; Figure S6—Decolorization/photodegradation assay of In_2_S_3_:MO [10:1] at pH 5; and Figure S7—Full-range UV–vis spectra of nanophotocatalyst ZnS/MB [45:1] at pH 5. Table S1—Summary of R^2^ values for pseudo-first-order (PFO) and pseudo-second-order (PSO) mathematical model (PDF)
