# Supplementary material for: Eco-Friendly Carboxymethyl Cellulose-Functionalized Binary (ZnS, In2S3) and Ternary (Zn–In–S) Quantum Dot Nanophotocatalysts for the Advanced Photodegradation of Organic Dye Pollutants in Water
Source: ACS Omega. 2026 Jul 1;11(27):39665–88. doi: 10.1021/acsomega.5c12542 (PMC13382682; doi:10.1021/acsomega.5c12542)
Supplement: Supplementary file 1 [file ao5c12542_si_001.pdf]

Supporting Information

Eco-Friendly Carboxymethyl Cellulose-Functionalized Binary (ZnS, In_2S_3) and Ternary (Zn-In-S) Quantum Dot Nanophotocatalysts for the Advanced Photodegradation of Organic Dye Pollutants in Water

Thiago Luís de Souza Esteves[1], Alexandra A. P. Mansur[1], Isadora C. Carvalho[1**], Herman S. Mansur[1,*]

[1] *Center of Nanoscience, Nanotechnology, and Innovation – CeNano²I, Department of Metallurgical and Materials Engineering, Federal University of Minas Gerais, UFMG, Av. Pres. Antônio Carlos, 6627 – Engineering School, 31.270-901, Belo Horizonte/MG, Brazil.*
*E-mail: *hmansur@demet.ufmg.br, **isadoracota@demet.ufmg.br*

Supplementary Figures

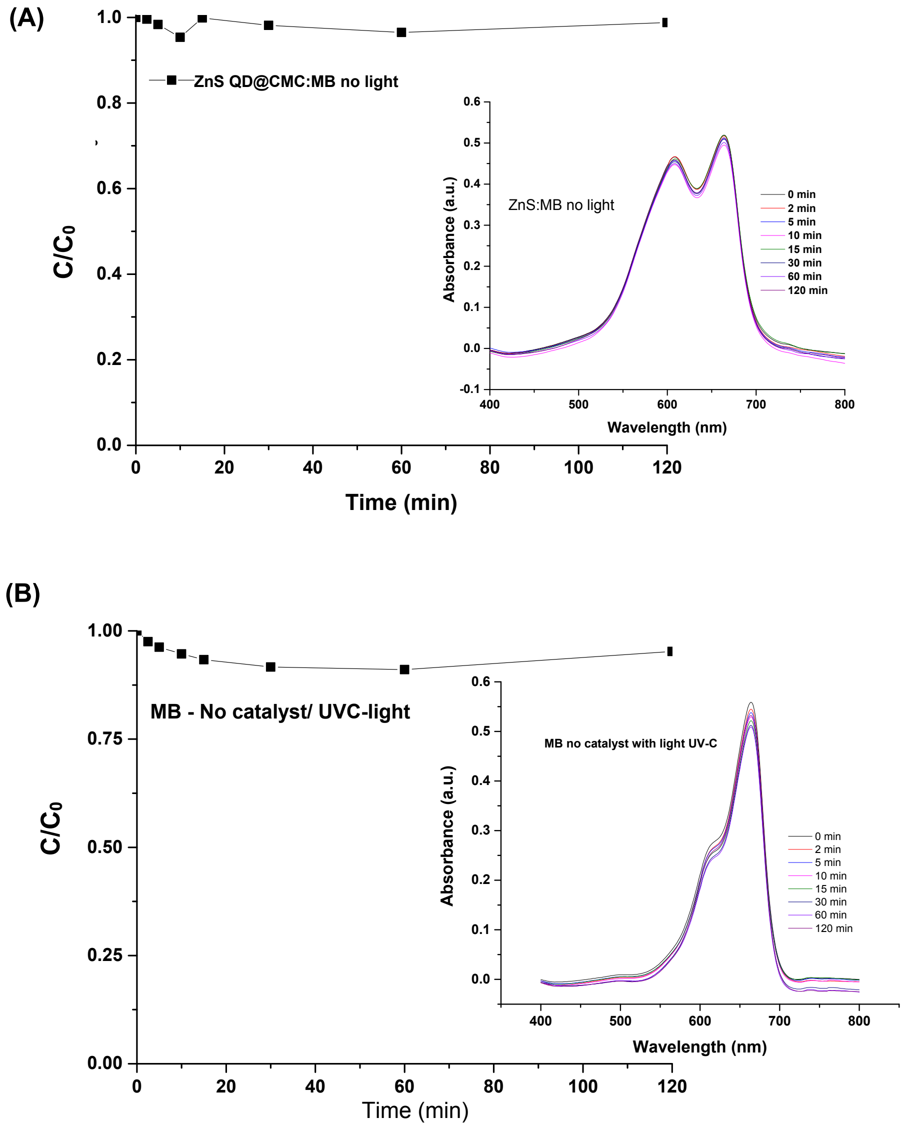

Figure S1 - Controls of catalysis assays (pH 5). (A) Test with ZnS:MB without irradiation ("dark"; inset: UV-vis spectra evolution with time). (B) MB in the absence of nanocatalysts and with irradiation ("light"; inset: UV-vis spectra evolution with time).

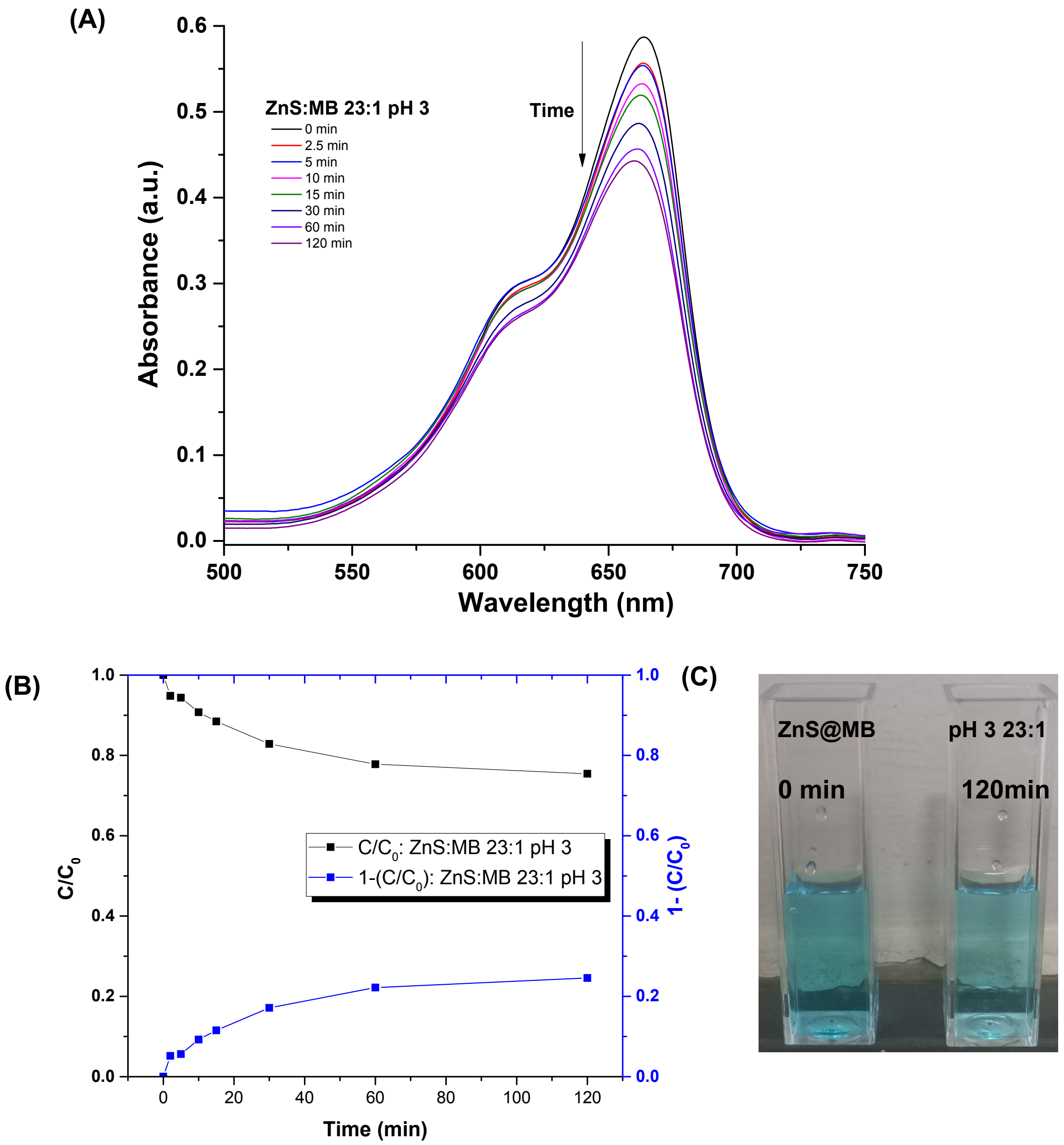

Figure S2 – Decolorization (i.e., photodegradation) assay of MB with nanophotocatalyst ZnS@CMC, ZnS:Dye [23:1] at pH 3. (A) UV-vis curves with time. (B) C/C_0 curve and degradation efficiency $(1-(C/C_0))$. (C) Digital Images at t=0 and 120 min of irradiation of samples.

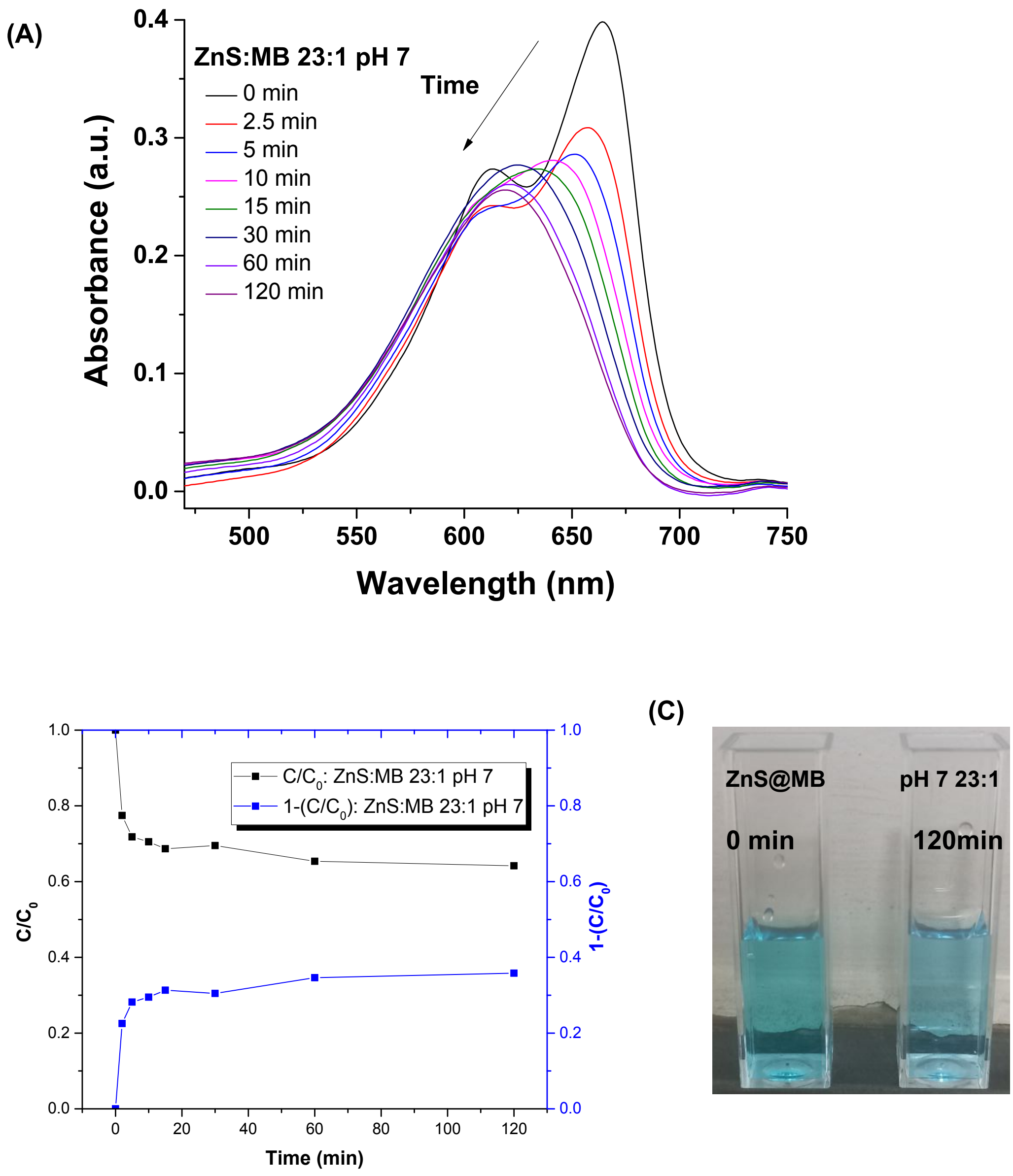

Figure S3 - Decolorization (i.e., photodegradation) assay of MB with nanophotocatalyst ZnS@CMC, ZnS:Dye [23:1] at pH 7. (A) UV-vis curves with time. (B) C/C_0 curve and degradation efficiency $(1-(C/C_0))$. (C) Digital Images at t=0 and 120 min of irradiation of samples.

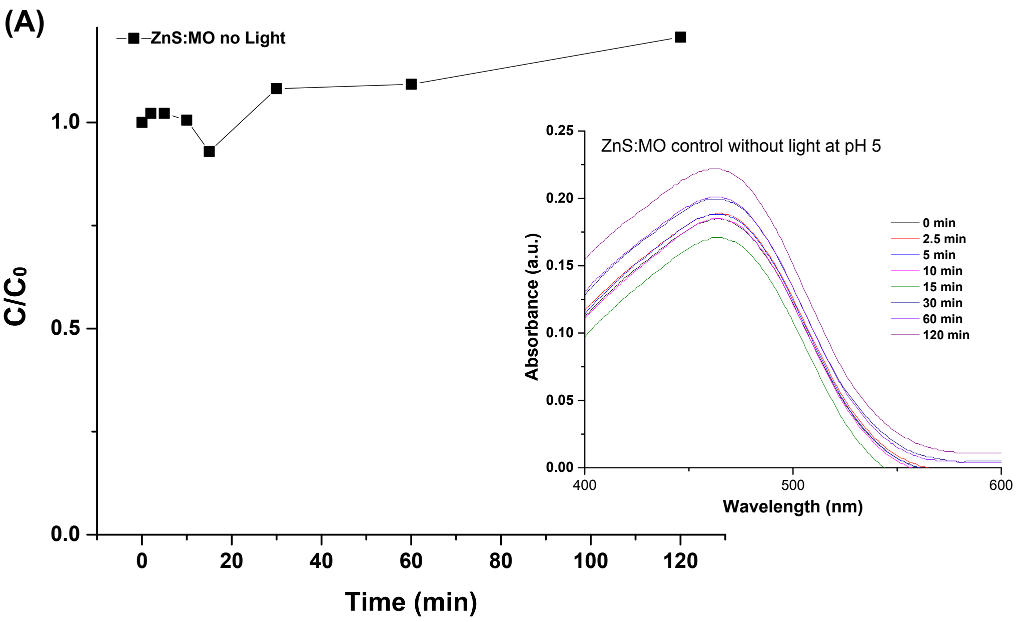

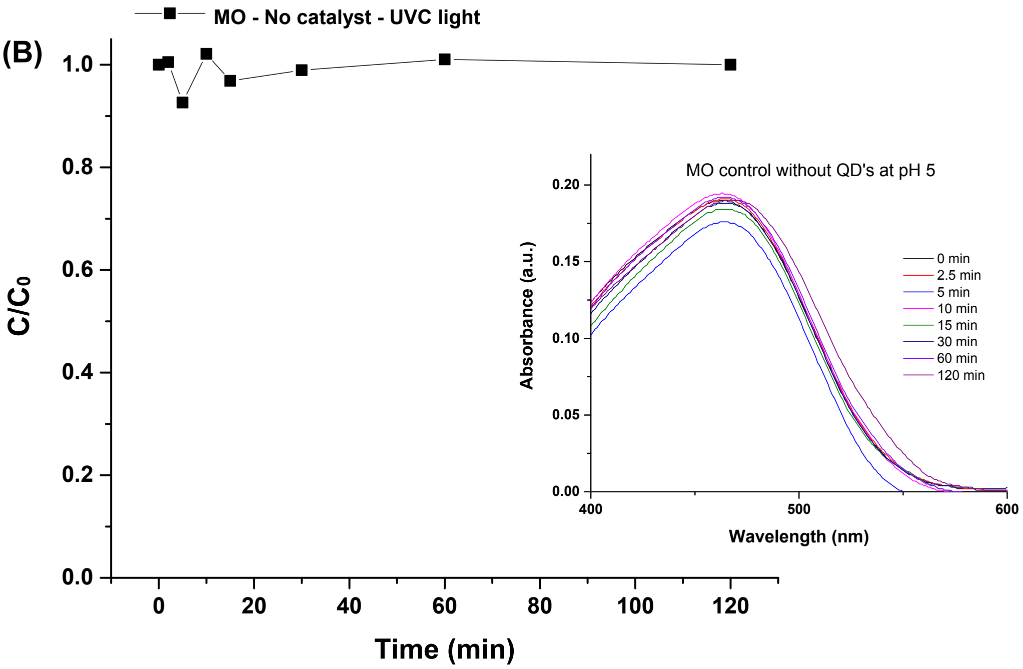

Figure S4 – Controls of catalysis assays (pH 5). (A) Test with ZnS@CMC:MO without irradiation ("dark"; inset: UV-vis spectra evolution with time). (B) MO in the absence of nanocatalysts and with irradiation ("light"; inset: UV-vis spectra evolution with time).

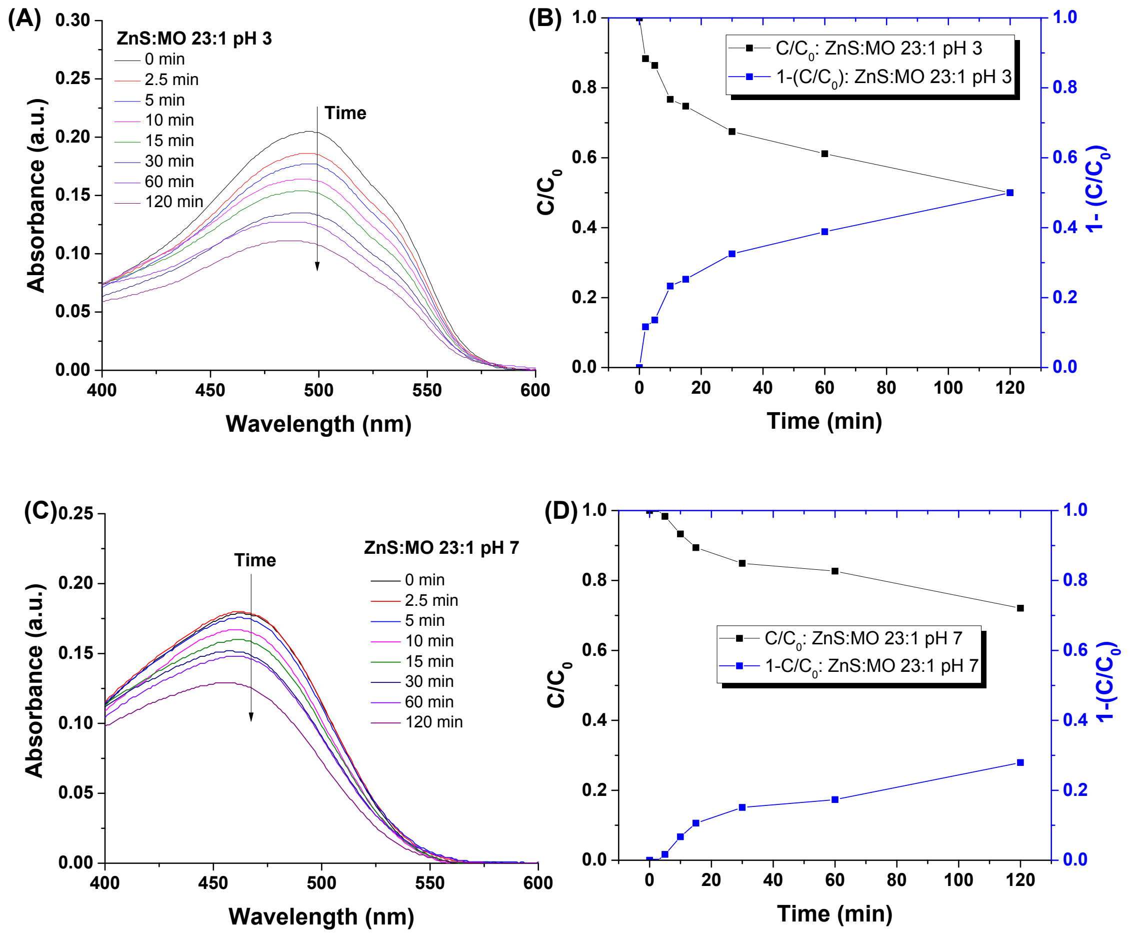

Figure S5 - Decolorization (i.e., photodegradation) assay of MO with nanophotocatalyst ZnS@CMC, ZnS:Dye [23:1] at pH 3 (A, B) and pH 7 (C, D). (A, C) UV-vis curves with time. (B, D) C/C_0 curve and degradation efficiency (1-(C/C_0).

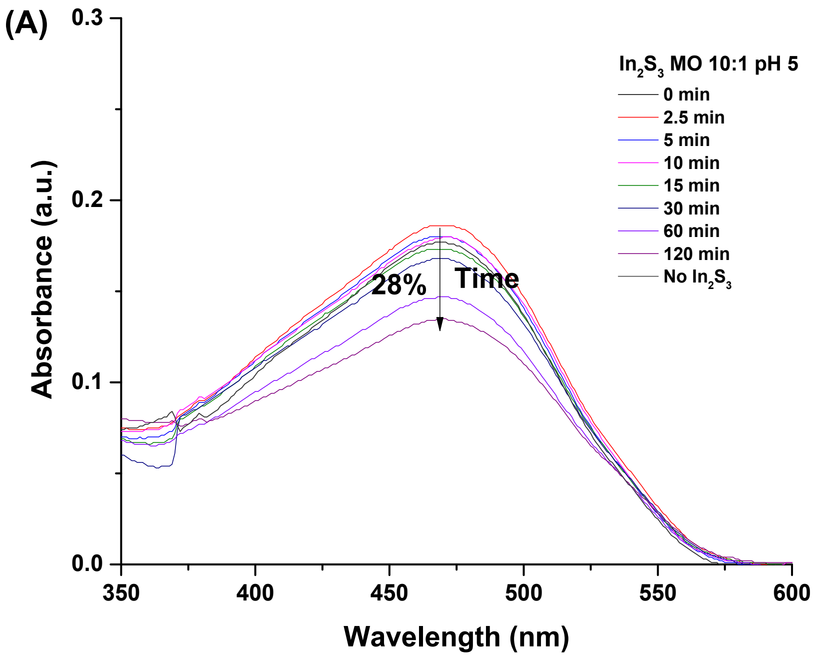

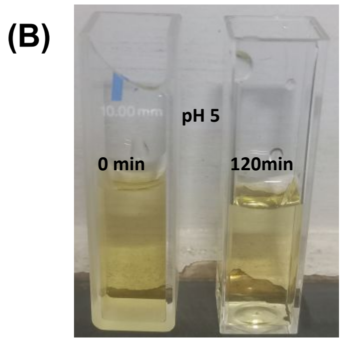

Figure S6 - Decolorization (i.e., photodegradation) assay of MO with nanophotocatalyst In_2S_3@CMC, In_2S_3:Dye [10:1] at pH 5. (A) UV-vis curves with time. (B) Digital Images at t=0 and 120 min of irradiation of samples.

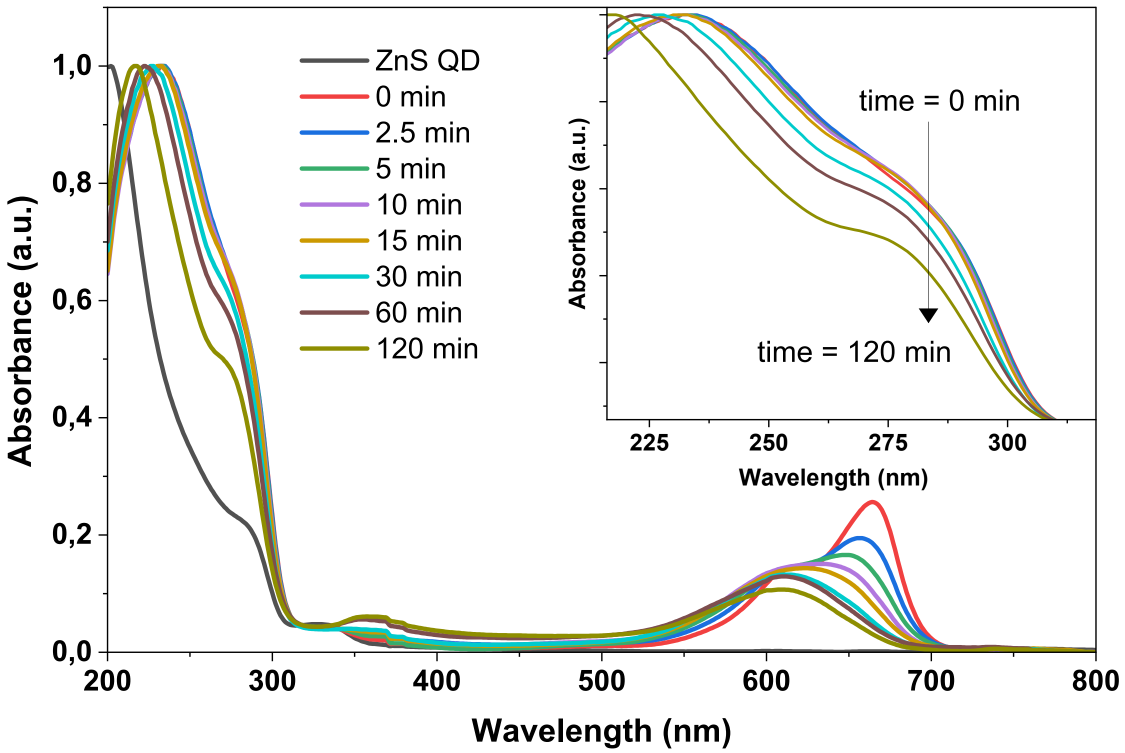

Figure S7 – Full-range UV-Vis spectra of nanophotocatalyst ZnS@CMC, ZnS:MB [45:1] at pH 5 (inset: detail of UV region of spectra).

Supplementary Table

Table S1 – Summary of R^2 values for pseudo-first-order (PFO) and pseudo-second-order (PSO) mathematical models for the degradation of organic dyes by photocatalysis based on ZnS@CMC nanocatalyst.

Dye	ZnS:Dye	pH	PFO	PSO
Methylene Blue (MB)	23:1	3	0.984	0.994
	23:1	5	0.982	0.999
	45:1		0.955	0.991
	23:1	7	0.964	0.986
Methyl Orange (MO)	23:1	3	0.975	0.993
	23:1	5	0.990	0.997
	23:1	7	0.983	0.984
